# Spatial structure, parameter nonlinearity, and intelligent algorithms in constructing pedotransfer functions from large-scale soil legacy data

**DOI:** 10.1038/s41598-020-72018-2

**Published:** 2020-09-14

**Authors:** Poulamee Chakraborty, Bhabani S. Das, Hitesh B. Vasava, Niranjan Panigrahi, Priyabrata Santra

**Affiliations:** 1 Agricultural and Food Engineering Department, Indian Institute of Technology Kharagpur, Kharagpur, WB 721302 India; 2 Division of Natural Resources, ICAR-Central Arid Zone Research Institute, Jodhpur, Rajasthan 342003 India

**Keywords:** Agroecology, Environmental impact

## Abstract

Pedotransfer function (PTF) approach is a convenient way for estimating difficult-to-measure soil properties from basic soil data. Typically, PTFs are developed using a large number of samples collected from small (regional) areas for training and testing a predictive model. National soil legacy databases offer an opportunity to provide soil data for developing PTFs although legacy data are sparsely distributed covering large areas. Here, we examined the Indian soil legacy (ISL) database to select a comprehensive training dataset for estimating cation exchange capacity (CEC) as a test case in the PTF approach. Geostatistical and correlation analyses showed that legacy data entail diverse spatial and correlation structure needed in building robust PTFs. Through non-linear correlation measures and intelligent predictive algorithms, we developed a methodology to extract an efficient training dataset from the ISL data for estimating CEC with high prediction accuracy. The selected training data had comparable spatial variation and nonlinearity in parameters for training and test datasets. Thus, we identified specific indicators for constructing robust PTFs from legacy data. Our results open a new avenue to use large volume of existing soil legacy data for developing region-specific PTFs without the need for collecting new soil data.

## Introduction

Soil information systems are increasingly used in developing ecosystem-scale understanding of critical zone processes and ecosystem services^1,2^. More recently, a greater role is attributed to large-scale soil data for realizing the sustainable development goals of food security, water management, and other health threats^3^. Comprehensive databases have been used to develop pedotransfer functions (PTF) for critical soils parameters such as soil hydraulic properties (UNSODA^4^, HYPRES^5^), soil organic carbon (SOC) contents (LUCAS^6^), and geochemical parameters (GEMAS^7^). Similarly, soil survey efforts have led to the creation of large repositories of legacy soil databases in many countries. Recently, legacy data for 196,498 geo-referenced locations covering 173 countries have been pooled to create a global soil information system under the umbrella of Global Soil Partnership^8^. Interestingly, much of legacy soil data remain largely underutilized^9^.

Although PTFs are attractive, their reliability depends on the amount (data size) and structure of the input parameters^10,11^. For instance, datasets with a few soil samples may be sufficient to develop reliable PTFs for relatively small geographical areas^12^. However, in large and heterogeneous landscape with high spatial soil variability^13^, PTF performance is influenced by the size and spread of soil sampling locations^14^. It is generally argued that PTFs should not be extrapolated beyond the geographical region or soil type from which they are developed^15–19^. Such a proposition has led to the creation of several region-specific and PTF-specific soil databases in countries and continents^4–7^. Ideally, the similarities or differences between the calibration and validation data and the underlying correlation structure should be considered as key determinants for the efficacy of a developed PTF^20,21^ rather than their geographical origin. Such a hypothesis has not been tested with experimental data to our knowledge. Specifically, what constitute key components of a training dataset and how to generate such a dataset are not clearly defined.

Spatial variability in soils is complex and soil properties generally do not follow spatial stationarity rules^22^. Moreover, many soil properties in samples collected from large areas show inherent non-linearities^14^. Legacy soil data also carry information on both spatial variability and non-linearity^23^. In addition, the size and volume of data available in many legacy soil databases are large^24^. Thus, legacy data may serve as a rich data source for developing region-specific PTFs if key features of a training dataset are well-defined and a methodology to extract such a dataset from legacy data is developed. Importantly, such a methodology will save time and effort needed in creating new datasets for developing region-specific PTFs.

**Figure 1:**
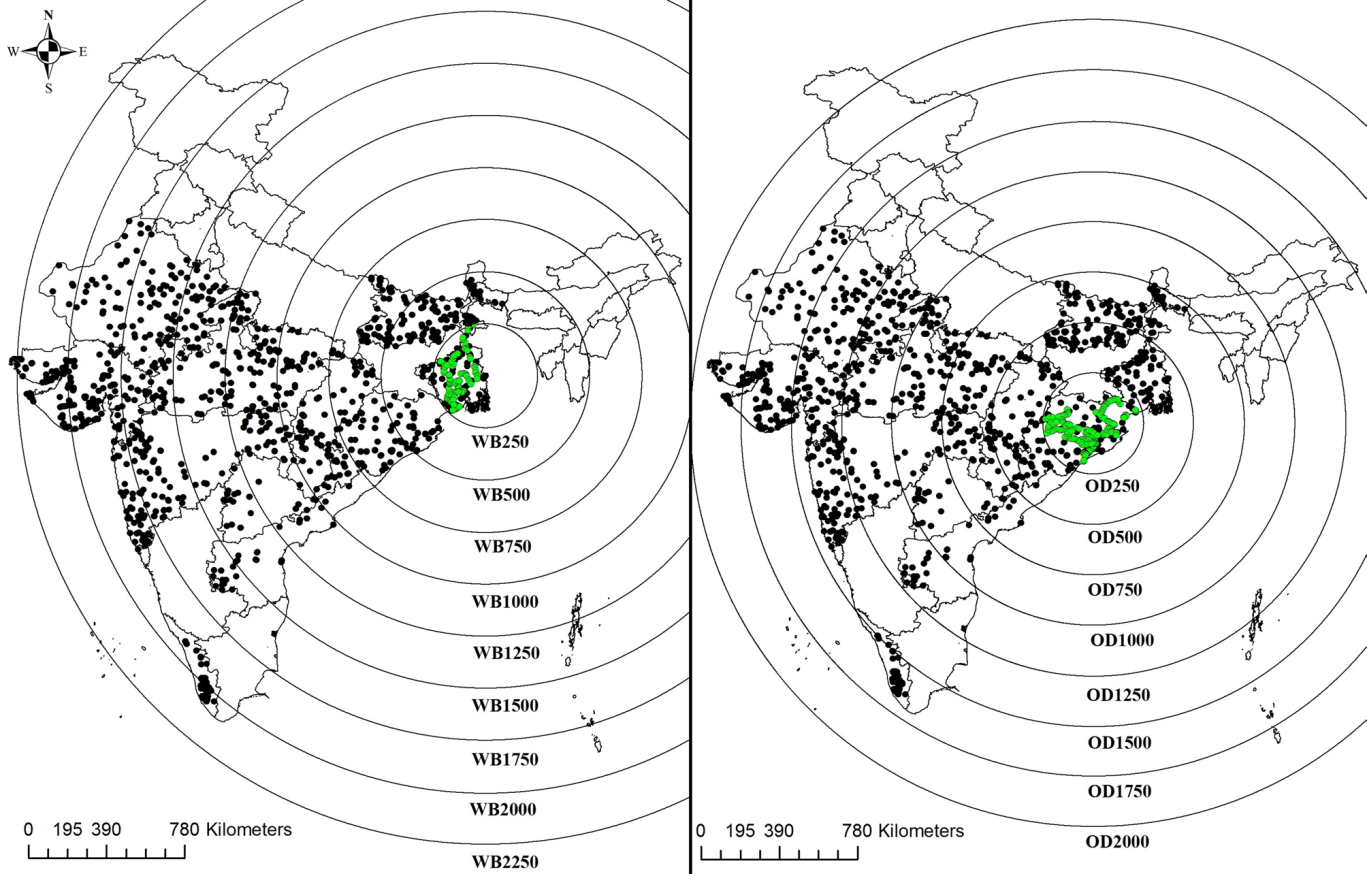
Map of India with the sampling locations for the Indian soil legacy data. Nine soil location datasets (WB250 to WB2250) were obtained by drawing circles with indicated radii of 250–2,250 km from the centre point (88.901°E and 23.126°N) located within the West Bengal state(right panel). Eight soil location datasets (OD250 to OD2250) were obtained by drawing circles with indicated radii of 250–2000 km from the centre point (85.584°E and 21.088°N) located within the Odisha state (left panel). The sampling locations for the West Bengal (WBT) and Odisha test data (ODT) collected by the Soil Physics laboratory at the Indian Institute of Technology Kharagpur, India is shown as green dots.

Therefore, the overall objective of this study was to examine if the large-scale legacy soil databases may be used for obtaining training data to calibrate PTFs. The specific objective was to examine how correlation structure, spatial variability, and non-linearity in training and test datasets influence PTF performance. To test these objectives, we selected the soil survey data collected by the National Bureau of Soil Survey and Land Use Planning (NBSS&LUP), Nagpur, India (hereinafter, referred to as Indian Soil Legacy or ISL database) as the legacy data source. We used the ISL database (shown with black dots in Fig. 1) for developing multiple training datasets for calibrating PTFs. Over the last decade, we also developed soil databases for the states of Odisha and West Bengal (shown with green dots in Fig. 1) as a part of building a spectral library for eastern Indian soils^25,26^. These two regional soil databases were used as independent test datasets; hereinafter, these databases are referred to as West Bengal test data (WBT database) and Odisha test data (ODT database). Because cation exchange capacity (CEC) data were available in all the three datasets and it is an important soil function parameter^27^, we selected CEC as a candidate for developing PTFs from legacy database. Both linear and non-linear modelling approaches such as multiple linear regression (MLR), ridge regression (RR), support vector regression (SVR), random forest (RF), and extreme gradient boosting (XGB) were examined to develop robust PTF for CEC. The XGB approach is an efficient machine learning algorithm^28^ and has not been used for developing PTFs in soil literature.

## Indian soil legacy database (training data)

The ISL database contained soil data for 4,190 soil horizons representing 1,092 georeferenced soil profile locations (Fig. 1) distributed over 11 Indian states: Andhra Pradesh^29^, Bihar^30^, Rajasthan^31^, West Bengal^32^, Madhya Pradesh^33^, Odisha^34^, Maharashtra^35^, Telangana^29^, Chhattisgarh^33^, Gujarat^36^, and Kerala^37^. These 11 states together occupy1.94 million km^2^ area and account for 59% of total geographical area of India. The soil sampling locations encompass four major cratonic provinces of India: Singhbhum in eastern India, Bastar in central India, Dharwar in southern India, and Aravalli–Bundelkhand in western India^38^. While granite-gneisses dominate Singhbhum craton, Dharwar and Aravalli–Bundelkhand are majorly basaltic in nature. Prevailing high rainfall and high temperature with granitic parent material in the eastern Indian regions have led to the formation of moderate to strongly weathered soils^25^ with the dominance of kaolinitic clays with different intergrades of smectites^39^. In contrast, basaltic parent material and low rainfall in the western Indian states have led to the formation of black cotton soils with vermicullitic and illitic clays in the Dharwar region^40^ while the deserts in the far western Rajasthan have very weakly developed soil profiles^41^. Thus, the ISL dataset has a wide variation in clay mineralogy and resulting CEC values and serves as rich pool of soil data for calibrating PTFs albeit the size of the dataset is still small compared to the total geographical area from which it is developed.

## West Bengal and Odisha database (test data)

Similar to variations in the ISL dataset, soils samples in the WBT and ODT datasets also encompass large variability because of contrasting geomorphological conditions. The WBT sampling locations were distributed in the whole of West Bengal state while the ODT sampling locations were distributed mostly along the four river systems (Subarnarekha, Brahmani, Baitarani and Mahanadi) of northern Odisha. Soils of West Bengal are classified into five soil chrono-associations^42^: Ganga Floodplain (age: 0.5 ka), Bhagirathi Plain and Old Ganga Plain (age: 1–1.5 ka), Barind Tract (Lower Level) and Damodar Deltaic Plain (age: 3–4 ka), Bhagirathi-Ajay Plain and Ajay-Silai Plain (age: 5–6 ka), and Upland with Red Soils (age: 350–1,000 ka). Such detailed geomorphological studies are not available for ODT samples. Nevertheless, there is a large variation in elevations in northern part of Odisha because of the presence of both plain and mountainous landscape. The elevation at the sampling locations of WBT database ranged from 3 to 156 m above the mean sea level (MSL) whereas those of ODT samples ranged from 1 to 589 m above MSL (the elevation map for the two states is provided in the Supplementary Document as Fig. S1). The parent material in the ODT samples are primarily of Archean and Proterozoic age^43^. Soils of both these sites show moderate to high weathering intensities with slightly higher silica to sesquioxide molar ratios and lower K_2_O contents for the ODT than WBT sampling locations^25^. They majorly represent Alfisols, Inceptisols, and Entisols with limited samples categorized under the Ultisol and Oxisol soil orders^40^ (USDA soil classification). With differing landforms and similar weathering stages, these two soil datasets may be treated as independent test data for examining the performance of developed PTFs.

## Selection of training data from large-scale soil legacy database

### Locational similarity

We used a series of preliminary modelling to identify an appropriate subset of training data from the ISL database. First, we used the ISL data from West Bengal and Odisha state as training datasets because of their similarity with the test datasets (WBT and ODT) in terms of geographical origin. Although we had 438 soil records in West Bengal and 432 soil records for the Odisha subset of ISL data, resulting PTFs failed to perform well for estimating the CEC values in both the test datasets. We also used the environmental covariates (i.e., elevation, average precipitation, and average temperature) for both these states as predictors of CEC along with the clay content, SOC content, and pH values; resulting PTFs under-predicted the CEC values for the respective test datasets. These results suggested that the local soil legacy data alone are inadequate for developing robust PTFs contradicting the long-standing notion that the training and test datasets for PTFs should be drawn from the same geographical region^16–18^. To test this observation further, we used the k-mean clustering approach to divide the entire ISL database into distinct (similar) clusters based on clay content, pH, and SOC triplets. Resulting PTFs developed with these clusters and their combinations did not describe the variability in CEC values in test datasets. Clustering approach created training datasets that had soil locations randomly scattered over the entire study area (covering all the 11 states). Consequently, the clusters and their combinations lacked inherent spatial correlation for the predictor and response soil properties. Moreover, local pedogenetic environment is known to influence soil characteristics (clay and SOC contents) linked to cation exchange behaviour. For instance, the eastern Indian soils have a high weathering intensity^25^ leading into dominantly kaolinitic clay mineralogy, which would impart characteristically low CEC values. Therefore, we concluded that locational similarity between training and test datasets should form an important criterion in developing PTFs in addition to other features in training datasets. To explore the later, we examined the ISL data beyond the geographical origin of test datasets and examined the variability and nonlinearity in soil properties, which may contribute to the performance of a local PTF.

### Spatial structure in training and test data

For selecting training data beyond locational similarity with test data, we examined spatial structures in the ISL and test datasets. The geometric extent (maximum width of a spatial data) for test datasets were more or less similar. For instance, a circle with a radius of 237 km could enclose sampling locations in the WBT samples around the centre point of 88.901°E and 23.126°N located within the West Bengal. Similarly, the sampling locations in ODT samples could be enclosed with a radius of 242 km around the centre point of 85.584°E and 21.088°N located within Odisha. As Fig. 1 shows, all the sampling locations of legacy and test data may be enclosed within a circle of radius 2,250 km. Thus, the geometric extent in our study varies from 250 × 2 = 500 km for the test datasets to 2,250 × 2 = 4,500 km in the ISL dataset. Because both support and spacing for the dataset could not be varied, we chose to examine the spatial structure in the ISL dataset from geometric consideration by incrementally changing geometric extent from a radius of 250–2,250 km as shown by the concentric circles enclosing various sampling locations. Thus, we obtained 9 training datasets for West Bengal-centric data and 8 datasets for Odisha-centric data. The increment of 250 km was chosen for simplicity because the sampling locations in the test datasets could be enclosed by circles with radii of 237 km and 242 km for West Bengal and Odisha, respectively. Because the total number of soil records in the ISL database was small (1,092 profiles with 4,190 soil layers) and our test samples (N = 402) were collected from top 20 cm soil depth, we calibrated PTFs by selecting training data by dividing ISL dataset into surface and whole profile soil datasets. Details of resulting 34 training datasets are presented in the method section.

Before analysing spatial structure and nonlinearity in soil parameters, we estimated the mean and the coefficients of variation (CV) values for CEC, pH, clay and SOC contents in all the datasets (Table 1). In general, average values for these soil properties for the surface datasets were significantly different from those of the whole profile datasets (*t*-test at 5% significance level). Similarly, the average values for different soil properties were significantly different across two test datasets except for clay contents. The average clay contents and pH values in both test samples were lower than those for ISL datasets; opposite trend was observed for SOC contents. Both test datasets showed similar CEC values with the averages of 17.82 cmol_c_ kg^−1^ for WBT samples and 20.5 cmol_c_ kg^−1^ for ODT samples. These CEC values are indicative of mixed clay mineralogy. Indeed, the presence of kaolinite, illite, and smectites clays in the ratio of 9:28:49 in alluvial soils and in 60:21:6 for red and laterite soils have been reported in West Bengal soils^39^. The training datasets showed a slightly wide variation in CEC with averages ranging from 11.91to 21.9 cmol_c_ kg^−1^. Larger CEC values for both test datasets than their local ISL training data (i.e., WB250 and OD250) suggest that the PTF derived with the regional training datasets may not always capture the variability in test data. The CV values for clay contents in the WBT and ODT samples were higher than those of the ISL location datasets; the opposite trend may be seen for the SOC and CEC values. Variations in all the four soil parameters are shown using box plots in Supplementary Document (Fig. S2).

**Table 1:** Mean and percentage coefficient of variation (given in parenthesis) for clay, pH, soil organic carbon (SOC) and cation exchange capacity (CEC) along with the number of data (N) for the test datasets and Indian soil legacy (ISL) location datasets obtained by taking a centre within West Bengal (WB) and Odisha (OD) states.

Location datasets	N		Clay content (%)		pH		SOC (%)		CEC (cmol_c_ Kg^-1^)	
	WB	OD	WB	OD	WB	OD	WB	OD	WB	OD
**Surface samples**										
Test	102	300	26.9 (51)	27.4 (41)	5.8 (15)	5.9 (15)	0.92 (36)	0.79 (39)	17.82 (52)	20.50 (47)
250	62	73	27.8 (50)	28.8 (44)	6.2 (12)	5.9 (16)	0.48 (48)	0.56 (88)	13.48 (60)	13.05 (73)
500	199	287	25.9 (46)	28.7 (43)	6.1 (12)	6.1 (12)	0.59 (108)	0.61 (80)	11.91 (63)	13.87 (61)
750	328	466	26.9 (44)	29.3 (43)	6.1 (13)	6.2 (13)	0.60 (95)	0.69 (87)	12.65 (60)	15.22 (68)
1,000	418	571	28.5 (42)	30.5 (43)	6.1 (13)	6.4 (13)	0.66 (92)	0.71 (81)	13.80 (64)	17.63 (71)
1,250	544	696	30.1 (42)	31.3 (44)	6.3 (13)	6.5 (14)	0.70 (82)	0.71 (85)	16.85 (69)	19.46 (70)
1,500	643	884	30.8 (44)	32.0 (45)	6.4 (14)	6.7 (14)	0.70 (78)	0.74 (83)	18.35 (71)	20.77 (69)
1,750	812	1,030	31.3 (45)	32.2 (46)	6.6 (15)	6.7 (17)	0.70 (84)	0.80 (99)	20.27 (70)	20.50 (72)
2000	1,000	1,092	32.3 (46)	32.2 (46)	6.7 (16)	6.7 (17)	0.77 (97)	0.79 (99)	20.90 (71)	20.71 (72)
2,250	1,092		32.2 (46)		6.7 (17)		0.79 (99)		20.71 (72)	
**Whole profile (surface + subsurface) samples**										
250	301	339	33.8 (43)	34.4 (37)	6.5 (12)	6.4 (14)	0.28 (67)	0.45 (196)	15.96 (53)	15.72 (64)
500	909	1,820	30.4 (45)	35.2 (38)	6.5 (12)	6.5 (12)	0.33 (125)	0.41 (157)	14.14 (66)	16.78 (59)
750	1,470	2,014	31.8 (43)	34.8 (41)	6.6 (13)	6.6 (13)	0.37 (149)	0.45 (135)	14.83 (61)	17.58 (66)
1,000	1,820	2,395	34.0 (41)	36.6 (41)	6.5 (13)	6.7 (13)	0.43 (144)	0.46 (127)	16.41 (63)	19.96 (67)
1,250	2,271	2,797	35.8 (39)	36.7 (41)	6.6 (13)	6.8 (13)	0.45 (131)	0.47 (122)	19.24 (65)	21.33 (66)
1,500	2,617	3,438	36.4 (40)	36.6 (43)	6.7 (13)	6.9 (14)	0.46 (123)	0.49 (121)	20.49 (66)	22.16 (66)
1,750	3,106	4,028	36.0 (42)	36.7 (42)	6.9 (14)	7.0 (21)	0.47 (127)	0.55 (244)	21.53 (66)	21.63 (69)
2000	3,810	4,190	36.6 (43)	36.6 (43)	6.9 (15)	6.9 (16)	0.52 (136)	0.54 (132)	21.90 (68)	21.55 (69)
2,250	4,190		36.6 (43)		6.9 (16)		0.54 (132)		21.55 (69)	

*Location dataset 250 correspond to soil samples collected from the area enclosed in a circle with 250 km radius from a centre point taken within West Bengal state (for those data included under column heading WB) and Odisha state (for those data included under column heading OD). The same is true for rest of the location datasets.

### Spatial variability in test data

The WBT dataset resulted in a linear semivariogram for CEC with nugget = 32.13, sill = 52.38, and range = 24.45 km while the ODT data showed a pure nugget variogram (nugget = 105.18). We repeated the semivariogram analyses by removing trend in the CEC data for the WBT samples and results showed a pure nugget effect similar to the ODT dataset. With no spatial structure, CEC values in test datasets may be considered as randomly distributed over the testing areas.

### Spatial variability in training data

**Figure 2:**
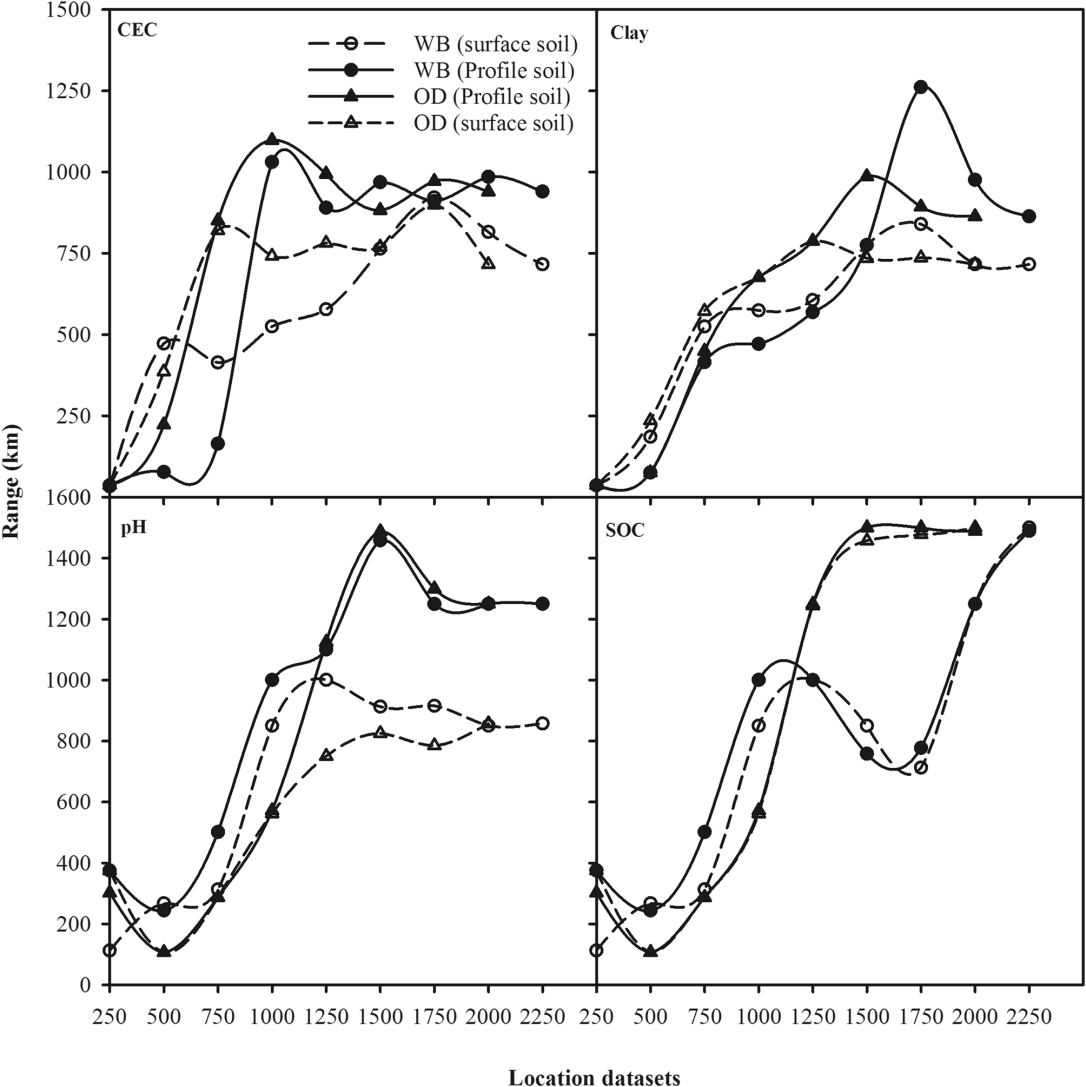
Ranges (km) for the theoretical semivariograms fitted to cation exchange capacity (CEC), clay, pH and soil organic carbon (SOC) for each West Bengal (WB) centric and Odisha (OD) centric location datasets vs. the radii (km) for each of these location datasets.

Similar to test datasets, we removed trends from all the 34 training datasets before fitting semivariograms. In general, a spherical model was fitted to the residuals of CEC, clay, and pH values while an exponential model was fitted to the residuals of SOC values. The range values for the semivariograms fitted to each of the soil properties for each of the training datasets are plotted as a function of the radius of the training dataset (Fig. 2). Figure 2 shows that training datasets have range values of about 1,250 km for CEC, 1,000 km clay, and about 1,500 km for pH and SOC values. With the range parameter varying from 1,000–1,500 km, one would expect to have spatially correlated response and predictor variable even if we use the entire ISL database as the training dataset. Semivariograms obtained for West Bengal centric training datasets of surface soils and Odisha centric training datasets for whole profile soils are shown for illustration as Supplementary Material (Fig. S3).

### Correlation structure among predictor and response variables

To examine the nature of relationships between CEC and its predictors in the ISL training datasets, we fitted generalized additive models (GAM) to predict CEC from clay content, pH, and SOC content. Spline-fitted pH, clay and SOC contents for the 9 West Bengal-centric and 8 Odisha-centric training datasets are presented in the Supplementary Documents (Fig. S4). The smoothing splines and their effective degrees of freedom (df) show that clay content had a stronger nonlinear relationship with CEC values for the whole profile soil samples than for surface soil samples. For surface soil samples, the extent of nonlinearity for clay contents was highest in the WB2000 (df = 8.45) and OD2000 (df = 8.34). The pH values had almost linear variations with CEC for surface soil samples up to 1,000 km radius in the West Bengal-centric and 500 km radius in the Odisha-centric datasets. Similarly, the SOC values had linear relationship with the CEC values in WB250, WB500, WB 750, and WB1250 calibration datasets for the surface soil group whereas WB1000, WB1500, WB1750, WB2000, and WB2250 datasets showed nonlinearity. For the whole profile soil samples, the SOC values showed linear relationship with the CEC values for WB250 and WB500 location dataset whereas nonlinear relationship was observed for the remaining location training datasets (Fig. S3). All the Odisha-centric training datasets showed nonlinearity for SOC values in both surface and whole profile soil groups. Interestingly, the observed the partial residuals for SOC for both Odisha and West Bengal training dataset compared to pH and clay content data (Fig. S4) suggested that SOC may not be a strongly contributing variable to CEC although model performance significantly changed when SOC was excluded in the PTF development (*t*-test at 1% significance level). This may be expected because the SOC contents for the ISL datasets are inherently low. The GAM analyses indicated that the extent of nonlinearity generally increases with the increase in the spatial extent of the soil datasets suggesting that a linear PTF model may be developed with soils belonging to the 250–750 km radius, however, as the spatial extent increases, a nonlinear modelling algorithm should be followed.

**Figure 3:**
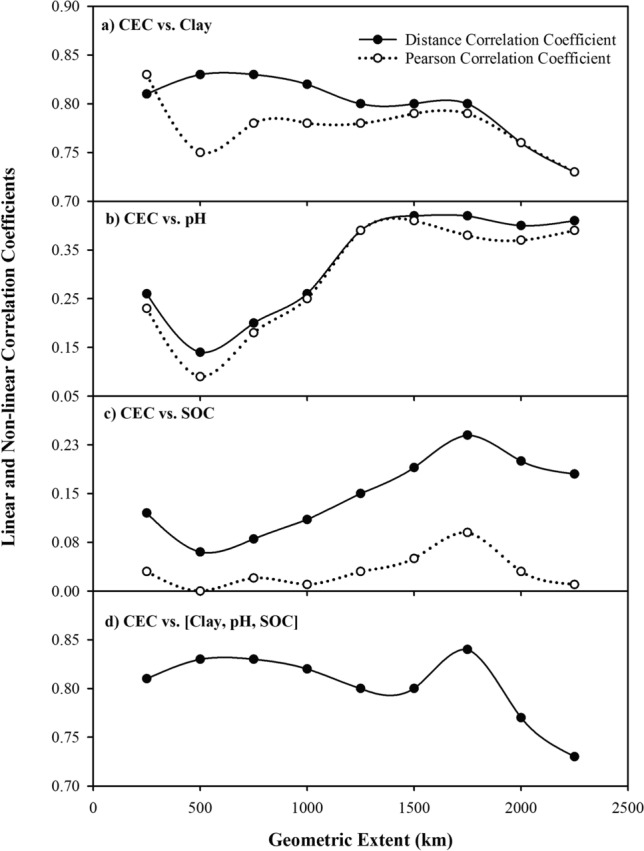
Pearsons correlation coefficient and distance correlation coefficient values between cation exchange capacity (CEC) and clay content (clay), CEC and pH , CEC and soil organic carbon content (SOC), and CEC and clay, pH, SOC combined for the West Bengal centric legacy location datasets for the whole profile soil samples.

With linear and nonlinear relationships between CEC and its predictors, the Pearson correlation coefficient (σ) and distance correlation coefficient (dCor) were estimated to quantify the extent of correlation between CEC and its predictor variables. Figure 3 shows the σ vs. dCor values for nine West Bengal-centric whole profile training datasets as an example; all the 238 different correlation coefficients are listed in Table S6 (Supplementary Material). In general, Fig. 3 shows that both the σ and dCor values for clay contents were higher than for the other predictor variables, pH and SOC content, because of the close relationship between clay types and CEC. Soil reaction appeared to be the next important predictor variable. With typically low SOC contents in Indian soils, the correlation coefficients between CEC and SOC contents were lowest in magnitude in all the training datasets. An important observation in Fig. 3 is that an increase in the contributing sampling area (from 250 to 2,250 km) altered the correlation coefficients for the individual predictors and CEC in different ways. The dCor values for clay content showed a general decreasing trend while the Pearson correlation coefficients first increased and then decreased with the increase in geometric extent (Fig. 3a). In contrast, both the coefficients for soil pH reached a minimum value at about 500 km and thereafter sharply increased before reaching a plateau at about 1,250 km (Fig. 3b). Soil organic carbon contents showed yet different trend by reaching a minimum value at about 500 km and a maximum value at about 1,750 km (Fig. 3c). When all the three variables were considered together, the dCor value showed a clear peak at 1,750 km (Fig. 3d) suggesting that three contributing predictor variables of clay, pH and SOC triplets for the datasets enclosed in a radius of 1,750 km will show maximum correlation with the CEC values compared to other training datasets. Similar results were observed for the remaining three datasets: High dCor values were reached for the datasets enclosed with 2000 km radius for the West Bengal- and Odisha-centric surface soil samples and with 1,500 km radius samples for Odisha-centric whole profile samples. Interestingly, the training datasets showing the highest dCor coefficient between CEC and SOC contents were identical to those showing the highest dCor coefficient between CEC and all the predictors combined. This clearly suggests that SOC content is a key parameter for estimating CECs in addition to clay contents. Thus, Fig. 3 shows that WB1750, WB2000, OD1500, and OD2000 may serve as the best training datasets*.* It may be noted that the consideration of calibration (training) data from regions enclosed within radii of 1,500 to 2000 km include soil samples developed from basaltic rocks that favours the formation of expanding clays, which may serve as a reason behind high dCor values in these training datasets. Moreover, the geostatistical analysis of the predictor and response soil properties show that even for radii beyond 1,500 km, the properties show spatial similarity.

## PTFs for CEC from legacy data

**Figure 4:**
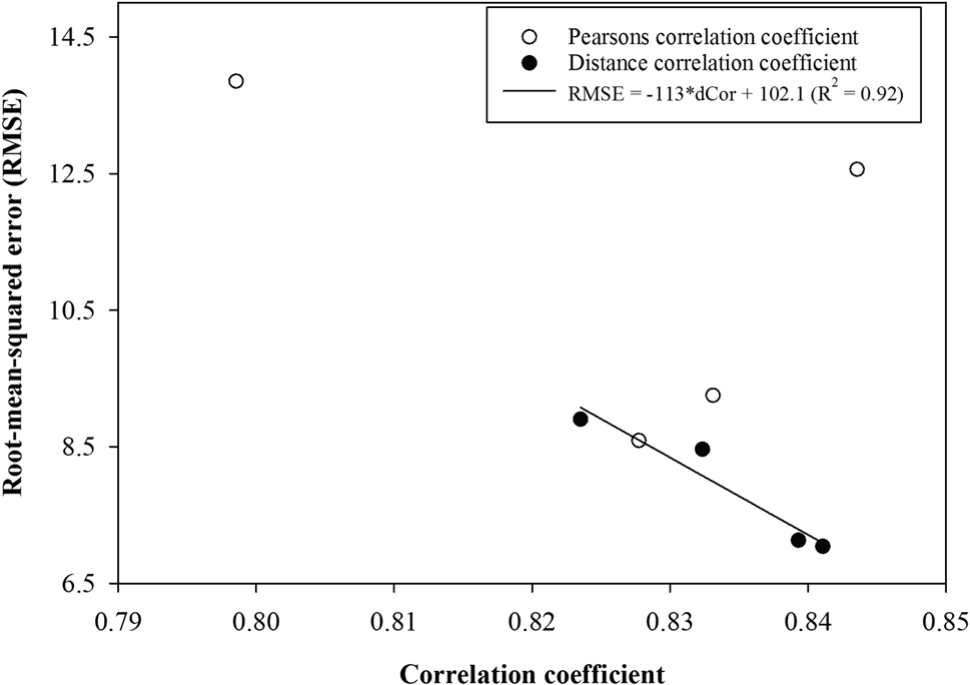
Maximum Pearsons correlation coefficients (ρ) vs. the minimum root mean squared error (RMSE) values for the PTFs tested on the West Bengal and Odisha test datasets trained on the specific training datasets that showed the maximum ρ. The figure also plots the maximum distance correlation coefficients (dCor) vs. the minimum root mean squared error (RMSE) values for the PTFs tested on the West Bengal and Odisha test datasets trained on the specific training datasets that showed the maximum dCor values.

Five different modelling approaches were used for 9 West Bengal-centric and 8 Odisha-centric ISL datasets and model performances were tested on WBT and ODT datasets. With separate surface and whole profile data treated as separate datasets, these modelling efforts led to having a total of 170 sets of R^2^ and RMSE values along with estimated bias (Tables 2 and 3). In general, the performance of the MLR- and RR-based PTFs decreased with the increase in the geographical extent of the training dataset because of the increase in the nonlinear relationship among response and predictor soil properties. Performance of the linear regression model in training datasets with small spatial extent may have resulted from the linearity in SOC and pH values observed for WB250, WB500, and WB750 datasets through GAM analyses. The effect of linearity on model performance may also be observed with generally lower R^2^ statistics for Odisha-centric than West Bengal-centric training datasets—GAM analyses suggested that relationships among predictor variables for CEC were more nonlinear and weaker for the Odisha-centric than West Bengal-centric training datasets. For the SVR approach, CEC values for the test datasets were over-predicted even when a genetic algorithm was used for tuning model parameters. The predictions by RF and XGB were almost similar; however, the boosting approach worked better than the bagging approach leading to superior performance of the XGB approach among all the five modelling algorithms used. Table 2 shows that the maximum validation (test) RMSE values of 7.13 cmol_c_ kg^−1^ and 7.04 cmol_c_kg^−1^ were observed in WB2000 surface and WB1750 whole profile training datasets, respectively. Similarly, the training datasets OD2000 (which is basically the entire ISL surface database) and OD1500 of the whole profile soil group provided the best prediction of CEC values for the ODT dataset with RMSEs of 8.46 cmol_c_ kg^−1^ and 8.90 cmol_c_ kg^−1^, respectively. Observed vs. predicted CEC values by the best PTFs trained on the best ISL training datasets are shown as supplementary Figure S5. Similar error values for predicting CEC using a single data source for subsampling calibration and validation datasets have been reported in the literature. For example, an RMSE value of 6.58 cmolc kg^−1^ was reported^44^. Therefore, we can conclude that we achieved a good prediction of CEC values for our independent test datasets. From the correlation structure between the response and predictor soil properties that we obtained, we chose the maximum ρ values. Interestingly, maximum ρ values were observed for CEC and clay contents in all the four training datasets (ρ = 0.83 in WB500 surface soils, ρ = 0.83 in WB250 whole profile soils, ρ = 0.84 in OD250 surface soils, and ρ = 0.80 in OD 250 whole profile soils). The RMSE values obtained for the prediction of CEC values using the datasets showing maximum ρ values and using MLR modelling approach were plotted in Fig. 4. In the same graph, we plotted the values of the minimum RMSE vs. the corresponding dCor values for the best training datasets (WB1750, WB2000, OD1500 and OD2000). No trend is observed for the ρ and RMSE values, however, a clear decreasing trend in the RMSE values with the increase in maximum dCor suggests that model performance is strongly controlled by parameter nonlinearity in the calibration (training) and validation (test) datasets.

**Table 2:** Coefficient of determination (R^2^), root-mean-squared error (RMSE) and bias values for the predicted cation exchange capacity (CEC) for the West Bengal test (WBT) dataset developed utilizing multiple linear regression (MLR), ridge regression (RR), support vector regression (SVR), random forest (RF) and extreme gradient boosting (XGB) modelling approaches trained on the Indian soil legacy (ISL) location datasets. WB250 to WB2250 location datasets correspond to soil samples collected from the area enclosed in a circle with radii from 250 km to 2,250 km and centre point at 88.901°E and 23.126°N as shown in Fig. 1.

Location	MLR			RR			SVR			RF			XGB		
datasets	R^2^	RMSE	Bias	R^2^	RMSE	Bias	R^2^	RMSE	Bias	R^2^	RMSE	Bias	R^2^	RMSE	Bias
**Surface soil samples**															
WB250	0.39	9.44	1.76	0.42	8.58	0.37	0.26	9.67	–4.94	0.31	8.84	–3.45	0.27	10.03	6.30
WB500	0.31	9.25	4.60	0.31	9.24	4.60	0.31	9.31	5.33	0.29	9.09	4.73	0.27	9.33	5.02
WB750	0.31	9.15	4.53	0.31	9.15	4.53	0.32	8.90	4.63	0.34	8.51	4.14	0.29	8.82	4.04
WB1000	0.31	9.35	4.69	0.32	9.31	4.69	0.32	8.98	4.70	0.35	8.46	4.13	0.43	8.09	4.16
WB1250	0.31	9.90	3.99	0.31	9.84	3.97	0.25	9.39	3.59	0.29	8.41	2.54	0.23	8.78	3.56
WB1500	0.31	10.25	3.80	0.31	10.17	3.77	0.27	9.24	3.27	0.32	8.24	2.30	0.37	7.99	2.35
WB1750	0.31	10.69	3.72	0.31	10.68	3.71	0.28	9.29	3.28	0.30	8.39	1.90	0.44	7.34	– 0.38
**WB2000**	0.32	10.46	4.33	0.33	10.39	4.29	0.29	9.01	3.47	0.30	8.38	1.98	**0.44**	**7.13**	**-0.02**
WB2250	0.33	10.45	4.64	0.34	10.32	4.56	0.29	9.00	3.55	0.33	8.17	1.91	0.41	7.30	1.95
**Whole profile (surface + subsurface) soil samples**															
WB250	0.35	8.59	4.09	0.35	8.60	4.14	0.27	10.34	6.72	0.33	9.24	5.27	0.28	9.85	6.06
WB500	0.32	9.07	4.61	0.32	9.07	4.62	0.30	9.40	5.23	0.25	9.32	4.25	0.23	9.45	3.51
WB750	0.32	9.07	4.61	0.32	9.06	4.63	0.30	9.40	5.23	0.26	9.23	4.40	0.23	9.45	3.51
WB1000	0.32	10.16	6.23	0.32	10.15	6.23	0.29	9.52	5.12	0.31	8.97	4.62	0.31	8.44	3.46
WB1250	0.31	10.54	6.08	0.31	10.49	6.04	0.27	9.44	4.07	0.26	8.86	3.41	0.39	7.44	2.14
WB1500	0.32	10.67	5.97	0.32	10.64	5.95	0.29	9.27	3.81	0.28	8.67	3.00	0.37	8.17	3.01
**WB1750**	0.33	10.87	5.89	0.33	10.82	5.86	0.29	9.31	3.61	0.30	8.47	2.43	**0.43**	**7.04**	**–0.44**
WB2000	0.34	11.36	7.02	0.34	11.31	6.98	0.33	8.97	4.33	0.32	8.41	2.78	0.43	7.33	2.22
WB2250	0.35	11.36	7.35	0.35	11.35	7.34	0.34	8.92	4.47	0.30	8.52	2.83	0.41	7.67	3.03

**Table 3:** Coefficient of determination (R^2^), root-mean-squared error (RMSE) and bias values for the predicted cation exchange capacity (CEC) for the Odisha test (ODT) dataset developed utilizing multiple linear regression (MLR), ridge regression (RR), support vector regression (SVR), random forest (RF) and extreme gradient boosting (XGB) modelling approaches trained on the Indian soil legacy (ISL) location datasets. OD250 to OD2000 location datasets correspond to soil samples collected from the area enclosed in a circle with radii from 250 to 2000 km and centre point at 85.584°E and 21.088°N as shown in Fig. 1.

Location	MLR			RR			SVR			RF			XGB		
datasets	R^2^	RMSE	Bias	R^2^	RMSE	Bias	R^2^	RMSE	Bias	R^2^	RMSE	Bias	R^2^	RMSE	Bias
**Surface soil samples**															
OD250	0.17	12.56	8.39	0.18	12.28	8.22	0.23	11.77	8.18	0.21	10.84	6.59	0.12	11.48	4.81
OD500	0.20	11.59	7.43	0.21	11.59	7.43	0.28	11.05	7.44	0.25	10.58	6.49	0.24	10.50	6.22
OD750	0.26	11.25	7.02	0.26	11.25	7.02	0.33	10.43	6.76	0.30	10.10	6.04	0.31	9.67	5.39
OD1000	0.28	11.13	6.37	0.28	11.11	6.36	0.34	10.26	6.38	0.31	9.72	5.39	0.30	9.07	4.16
OD1250	0.27	11.33	6.20	0.27	11.32	6.19	0.31	10.34	6.20	0.31	9.62	5.10	0.28	9.41	4.43
OD1500	0.27	11.26	6.00	0.27	11.23	5.97	0.34	10.03	5.85	0.30	9.46	4.39	0.30	9.23	3.88
OD1750	0.27	11.68	6.81	0.27	11.60	6.75	0.34	10.10	5.97	0.33	9.40	4.78	0.34	8.67	3.56
**OD2000**	0.26	11.68	6.76	0.26	11.62	6.71	0.34	10.11	6.00	0.34	9.39	4.79	**0.34**	**8.46**	**3.18**
**Whole profile (surface + subsurface) soil samples**															
OD250	0.11	13.85	9.78	0.11	13.84	9.78	0.23	11.16	7.17	0.14	11.63	7.26	0.17	11.50	7.58
OD500	0.20	12.56	8.90	0.20	12.54	8.89	0.30	11.06	7.55	0.22	11.06	7.04	0.26	10.59	6.59
OD750	0.25	12.32	8.63	0.25	12.29	8.61	0.32	10.85	7.29	0.29	10.60	6.81	0.28	10.10	5.89
OD1000	0.27	12.22	8.34	0.27	12.18	8.31	0.33	10.50	6.75	0.31	10.13	6.20	0.32	9.16	4.48
OD1250	0.28	12.35	8.34	0.28	12.31	8.30	0.31	10.50	6.55	0.32	9.92	5.84	0.28	9.47	4.68
**OD1500**	0.28	12.20	8.07	0.28	12.13	7.99	0.34	10.21	6.23	0.30	9.77	5.13	**0.32**	**8.90**	**3.19**
OD1750	0.25	13.15	9.41	0.24	11.47	7.42	0.32	10.80	6.96	0.29	10.22	5.90	0.30	9.28	4.46
OD2000	0.29	13.52	10.00	0.29	13.50	9.98	0.32	10.73	6.84	0.30	10.19	5.91	0.27	9.70	5.08

## Discussion

**Figure 5:**
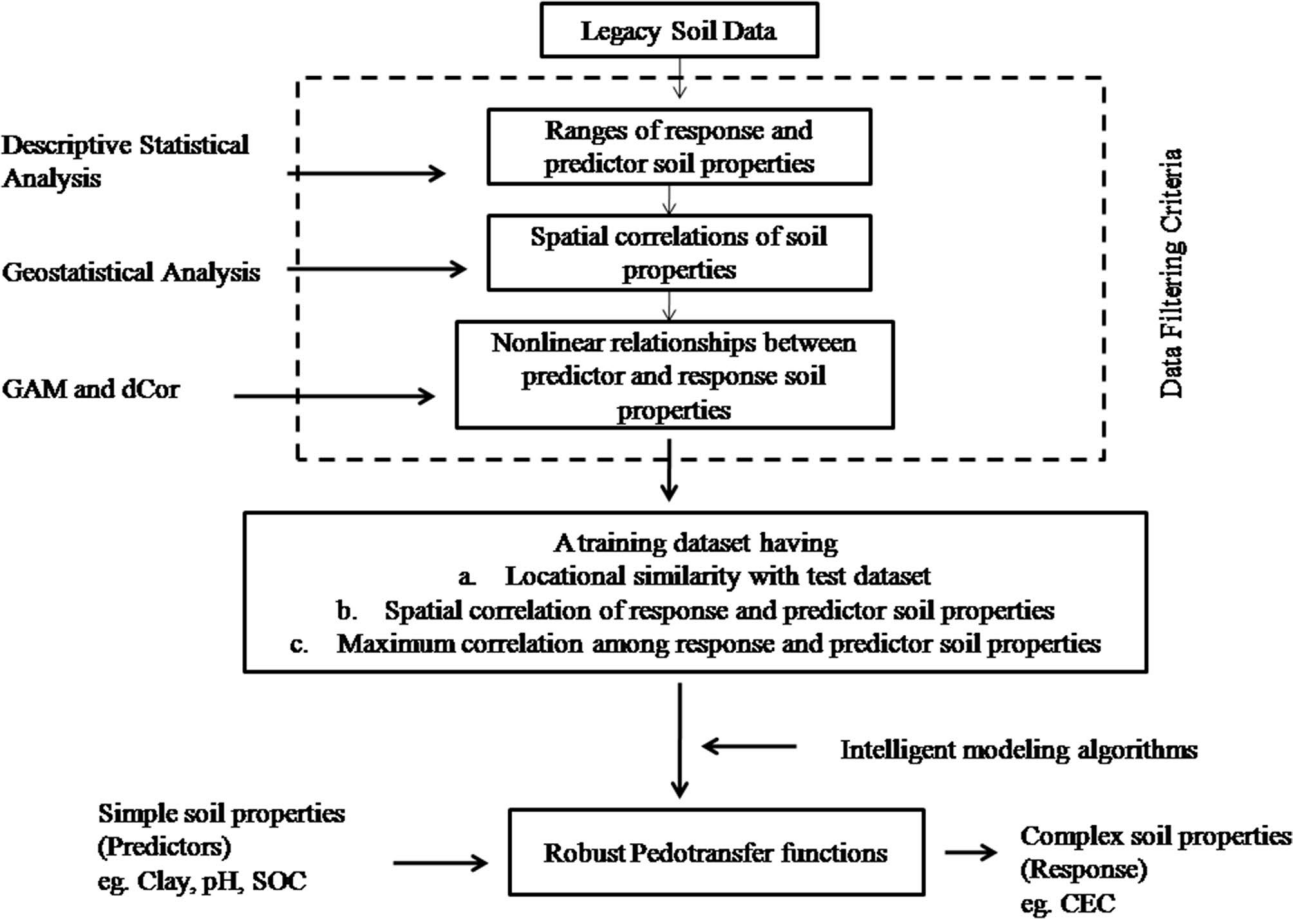
Scheme followed to utilize the legacy soil data to obtain region-specific robust pedotransfer functions for difficult-to-measure soil properties; GAM: general additive model, dCor: distance correlation, CEC: cation exchange capacity, SOC: soil organic carbon content.

Soil properties, soil functions, and PTFs are influenced by inherent nonlinearity of processes, spatial variability of soil parameters^45^, and diverse agro-climatic conditions. Collected from large areas, legacy data contain information on all these three attributes. Therefore, a carefully-selected subset of legacy data is sufficient to develop a robust regional PTF and, hence, identification of this subset is the key step in PTF analysis. With a series of modelling studies, we observed that the best training dataset had three critical attributes: (a) locational similarity between the training and test datasets, (b) the presence of spatial correlations for each of the predictor and response soil properties, and (c) the presence of a strong correlation between the predictor and response soil property. Indeed, PTFs developed with the legacy soil data belonging to the same location as that of the test data alone failed to predict the CEC values calling for additional features in training datasets. Combining soils randomly from multiple locations to develop a robust PTF might not work, because we observed that creating clusters from legacy data with locations spreading all over the country and creating PTF combining those clusters failed to predict the CEC for the test datasets. This also may be the reason that limits us to develop a PTF model using combined continental- and country-level legacy soil data. Indeed, we repeated our analysis by selecting training datasets around a centre point on roughly at the centre of the country (21.145°N, 79.088°E). Training datasets distributed with as wide as 1,000 km extent from such an India-centric centre point and the efficient XGB algorithm resulted in the R^2^ values of 0.30 (RMSE = 9.08) for WBT and 0.29 (RMSE = 9.33) for the ODT datasets. Such a result suggests that both training and test datasets should not only share locational similarity (as evident from clustering analysis), the predictor and response variables in the training datasets should have similar spatial structure. This requirement stems from the argument that the set of rules relating a response soil property with predictor properties may not become too much diverse if each soil property is spatially-correlated with itself. If such spatial dependency criterion is ignored, there may be chance of including sampling pairs having lag distance beyond range, where diversity may exist but the set of statistical rules relating predictor and response soil properties may differ reducing the performance of resulting PTFs. Therefore, a spatial correlation for the predictor and response soil properties forms the second criterion for an ideal training dataset. The third criterion results from the requirement for strong correlation structure between the predictor and response soil properties in a training dataset—both linear and nonlinear as may be inherent with specific soil properties. From the correlation structure analysed in our study, we observed that a nonlinear correlation coefficient is required to capture the inherent relationship among predictor and response soil properties. The final requirement pertains to the use of an intelligent predictive algorithm that exploits the three features of locational similarity in test and training datasets, spatial similarity among predictor and response variables, and inherent nonlinearity in soil parameters. We observed that the XGB algorithm showed best learning capability among all the predictive modelling approaches. Recently, it has also been shown that transfer rules learnt by the more general global models developed with the continental data on soil spectra were transferred to a local domain to generalize a soil spectral model^46^. A similar learning of the XGB model when trained on soils belonging to a wider geographical area as compared to the local soil samples might have helped in better performance of the developed PTFs in this study. These results are combined to build a scheme for choosing the training dataset from the legacy soil database in Fig. 5.The developed workflow may be used for deriving training datasets from legacy soil data. Specifically, the proposed criteria may be used to develop region-specific PTFs from legacy soil data in countries with a wide range in environmental and edaphic factors without collecting new soil data.

## Methods

### M1-collection and compilation of test dataset

The test datasets were generated in our laboratory as a part of building spectral library of soils for the eastern Indian states of West Bengal and Odisha during the last decade. We refer to these two state-wise databases as West Bengal Test (WBT) and Odisha Test (ODT) databases. The database consisted of surface soil samples available for 102 locations across West Bengal state and 300 locations across Odisha state, respectively (Fig. 1). For both the datasets, soil samples were collected mostly from agricultural fields cultivated with rice (*Oryza sativa* L.) crop. Collected samples were air‐dried and passed through a 2‐mm sieve before analysing them in laboratory to determine different soil properties. SOC contents for the processed soil samples were estimated using the chromic acid digestion method^47^, and particle size distribution was determined using the pipette method^48^. Soil pH was measured on a 1:2.5 soil:water suspension^49^. The CEC was determined by the ammonium saturation method at pH 7.0^50^. It may be noted that the ISL database also has soil data from both West Bengal and Odisha states. However, the sampling locations of those soil series are different from the sampling locations of soil samples present in the WBT and ODT databases (Fig. 1). Thus, the WBT and ODT databases serve as independent test datasets in our study.

### M2-selection of training datasets

Because we had only 4,190 records in the whole ISL dataset compared to the large region over which ISL data was developed, we chose to test our approach on ODT and WBT datasets separately. This provided two instances of model validation. To realize separate sets of training data for ODT and WBT test datasets, we considered generating both West Bengal-centric and Odisha-centric training datasets. We observed that the WBT data may be enclosed in a circle of radius 237 km with the centre located at 88.901°E latitude and 23.126°N longitude; similarly, the ODT data may be enclosed in a circle of radius 242 km with the centre located at 85.584°E latitude and 21.088°N longitude. For simplicity, we considered 250 km as the radius of the circle (instead of 237 or 242 km) located around the selected centre points for both the test data regions (Fig. 1). The ISL datasets located within these circles may be assumed to serve as local training data (i.e., having similar geographical origin as those of the respective test datasets). The furthest point along western India stretched about 2,250 km from the WBT and 2000 km from the ODT centre points. We arbitrarily divided these stretches of 2,250 km into concentric rings by drawing circles of radii 250, 500, 750, 1,000, 1,250, 1,500, 1,750, 2000, and 2,250 km and divided the ISL database into multiple location datasets. For example, the sampling points falling within 500 km from the centre point of 88.901°E and 23.126°N are considered location dataset corresponding to 500 km and so on. We refer to West Bengal-centric datasets as WB250, WB500, WB750,…, and WB2250. The number of soil samples belonging to each of these nine location datasets are 62, 199, 328, 418, 544, 643, 812, 1,000, and 1,092, respectively, when only surface soils of the ISL database is considered. When the whole soil profile is considered, the numbers of soils belonging to each of these location datasets are: 301, 909, 1,470, 1,820, 2,271, 2,617, 3,106, 3,810, and 4,190, respectively. Similarly, we obtained eight Odisha-centric location datasets (OD250, OD500, OD750,…, and OD2000). The number of soil samples belonging to each of these eight location datasets are 73, 287, 466, 571, 696, 884, 1,030, and 1,092, respectively when only surface samples of the ISL database is considered. When the whole soil profile is considered, the numbers of soils belonging to each of these location datasets are: 339, 1,280, 2014, 2,395, 2,797, 3,438, 4,028, and 4,190, respectively. Details of these location datasets (soil orders and agro-ecological zones to which these location datasets belong) are provided as Supplementary Documents (Table S7). Spatial analysis of data for visualization and sub-setting it in to different concentric zones were conducted with ArcGIS®(ESRI).

### M3-geostatistical analysis

We examined the spatial structure in all the training datasets and the test datasets for all the soil properties involved in the PTF development. For the whole soil profile data, weighted-average of the profile soil data was considered for each location for estimating the semivariograms. Different theoretical semivariogram functions were fitted to each of those experimental semivariograms to obtain the range, nugget, and sill values. These parameters provided an average measure of dissimilarity for a property as a function of separation distance. Best-fitted theoretical semivariogram models were chosen based on weighted least-square fitting where weights (w_i_) for each lag class were proportional to number of data pairs and inversely proportional to lag distance. As the soil samples were collected from wide geographical areas, we removed stationarity in the datasets before fitting the semivariogram models. A trend surface model was fitted to detrend the observed data using least-squared approach. The residuals (= difference between observed and modelled soil parameter) were then used to estimate semivariograms. All geostatistical analyses were carried out using the *lattice* and *gstat* packages in R programming environment^51^.

### M4-dependency measurements

A generalized additive modelling (GAM) approach^52^ was also used to examine the marginal relationship between CEC values and the predictor variable(s) such pH, clay and SOC contents. The basic idea in GAM is to fit a function on each of the predictors to capture relationships between the response and the predictor variables. The effective degrees of freedom of the smoothing spline fitted to each of the predictor variables is an indication of the underlying nonlinearity between the predictors and the response variable. A penalized smoothing spline approach was used to choose the effective degrees of freedom for the smoothing splines for this study using *mgcv* package in R programming environment^51^.

We used both linear and non-linear correlation measures for quantitatively assess the extent of correlation among different soil parameters. The Pearson correlation coefficient (ρ) describing linear correlation between two parameters is given as:

1 $$ \rho \left( {x,y} \right) = \frac{{n\left( {\sum xy} \right) - \left( {\sum x} \right)\left( {\sum y} \right)}}{{\sqrt {\left[ {n\sum x^{2} - \left( {\sum x} \right)^{2} } \right]\left[ {n\sum y^{2} - \left( {\sum y} \right)^{2} } \right]} }} $$

where *x* and *y* are two random variables and *n* is the number of variables. Similarly, the distance correlation^53^ (dCor) is a nonlinear dependency measure based on distribution or density functions and is given as:

2 $$ {\text{dCor}} = \frac{{dcov\left( {x,y} \right)}}{{\sqrt {dcov\left( {x,x} \right)dcov\left( {y,y} \right)} }} $$

where *x* and *y* are two random vectors. The distance covariance (*d*cov) function in Eq. (2) is estimated as:

3 $$ dcov^{2} \left( {x,y} \right) = \mathop \smallint \limits_{{R^{dx + dy} }}^{.}\| \phi_{x,y} \left( {t,s} \right) - \phi_{x} \left( t \right)\phi_{y} \left( s \right)\|^{2} w\left( {t,s} \right)dtds $$

where *ϕ*_*x*_*(t)* and *ϕ*_*y*_*(s)* are the respective characteristic functions of the random vectors *x* and *y*, *ϕ*_*x,y*_*(t, s)* is the joint characteristic function of x and y, and *d*_*x*_ and *d*_*y*_ are the dimensions of *x* and *y*, respectively. The weight function *w*(*t*, *s*) is given by

4 $$ w\left( {t,s} \right) = \left\{ {c_{{d_{x} }} c_{{d_{y} }}\| t\|_{{d_{x} }}^{{1 + d_{x} }} \|s\|_{{d_{y} }}^{{1 + d_{y} }} } \right\}^{ - 1} $$

with $c_{d} = \pi^{{\left( {1 + d} \right)/2}} /\left\{ {\left( {1 + d} \right)/2} \right\}$ .The dCor value between two variables is zero if and only if the two variables are independent^53^.

### M5-regression and data-mining approaches

Five different regression algorithms were used to develop PTFs for CEC: multiple linear regression (MLR), ridge regression (RR), support vector regression (SVR), random forests (RF), and extreme gradient boosting (XGB). We have considered an RBF kernel function and used genetic algorithm (GA) to optimize regularization parameter C, bandwidth of RBF kernel σ^2^ and radius of a tube loss function ɛ for the SVR model. Steps followed for optimizing SVR using GA is already reported^54^. For the RF-based model development, the number of trees in the forest (N_estimators) and the depth of each tree in the forest (max_depth) were tuned based on leave-one-out cross validation of the training dataset. For the XGB-based models, we tuned the learning rate, the maximum tree depth, fraction of observation to be randomly sampled, and fraction of column to be randomly sampled for each tree using a leave-one-out cross validation of the training datasets. We used *xgboost*, *e1071*, *randomForest*, *glmnet* library functions in R programming environment^51^ to implement the above modelling approaches. Details for each of these five modelling approaches are provided as Supplementary Document.

### M6-model accuracy

The accuracy of the models was adjudged using the coefficient of determination (R^2^), root-mean-squared error (RMSE), and bias values estimated as:

5 $$ R^{2} = 1 - \frac{{\mathop \sum \nolimits_{i = 1}^{N} \left( {Y_{i} - f\left( x \right)_{i} } \right)^{2} }}{{\mathop \sum \nolimits_{i = 1}^{N} \left( {Y_{i} - \overline{Y}_{i} } \right)^{2} }} $$

6 $$ RMSE = \sqrt {\frac{1}{N}\mathop \sum \limits_{i = 1}^{N} \left( {Y_{i} - f\left( x \right)_{i} } \right)^{2} } $$

7 $$ Bias = \frac{{\mathop \sum \nolimits_{i = 1}^{N} \left( {f\left( x \right)_{i} - Y_{i} } \right)}}{{\mathop \sum \nolimits_{i = 1}^{N} Y_{i} }} $$

where *N* is the number of samples, *Y*_*i*_is the observed value of the CEC, *f(x)*_i_ is the predicted value of CEC values.

## Supplementary information

Supplementary information
