# Supplementary material for: Spatial structure, parameter nonlinearity, and intelligent algorithms in constructing pedotransfer functions from large-scale soil legacy data
Source: Sci Rep. 2020 Sep 14;10:15050. doi: 10.1038/s41598-020-72018-2 (PMC7490350; doi:10.1038/s41598-020-72018-2)
Supplement: Supplementary file 1 — Supplementary information [file 41598_2020_72018_MOESM1_ESM.pdf]

SPATIAL STRUCTURE, PARAMETER NONLINEARITY, AND INTELLIGENT ALGORITHMS IN CONSTRUCTING PEDOTRANSFER FUNCTIONS FROM LARGE-SCALE SOIL LEGACY DATA

Poulamee Chakraborty*[a], Bhabani S. Das[a] , Hitesh. B. Vasava[a], Niranjan Panigrahi[a], Priyabrata Santra[b]

Supplementary Figures

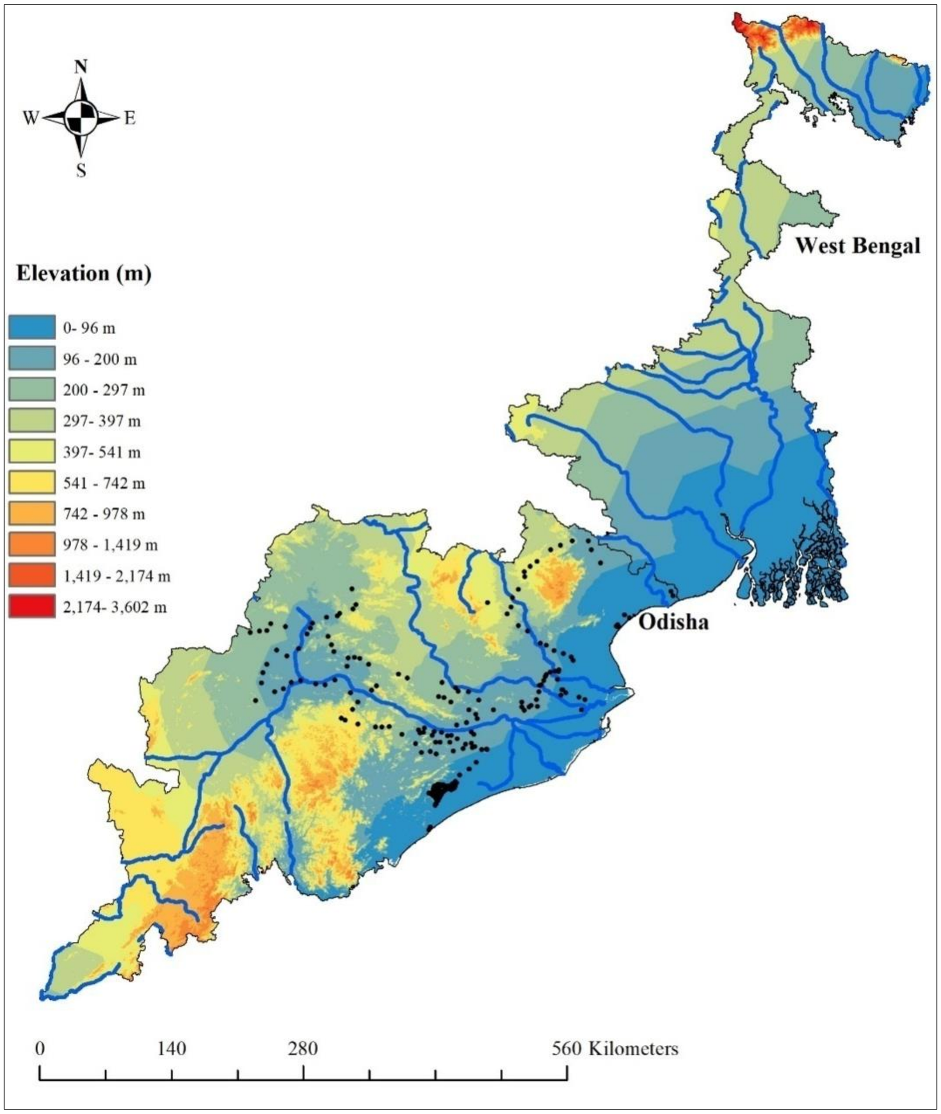

Fig. S1. Elevation above mean sea level in metres for the states of West Bengal and Odisha in Eastern India. The river network for the area is shown as blue lines

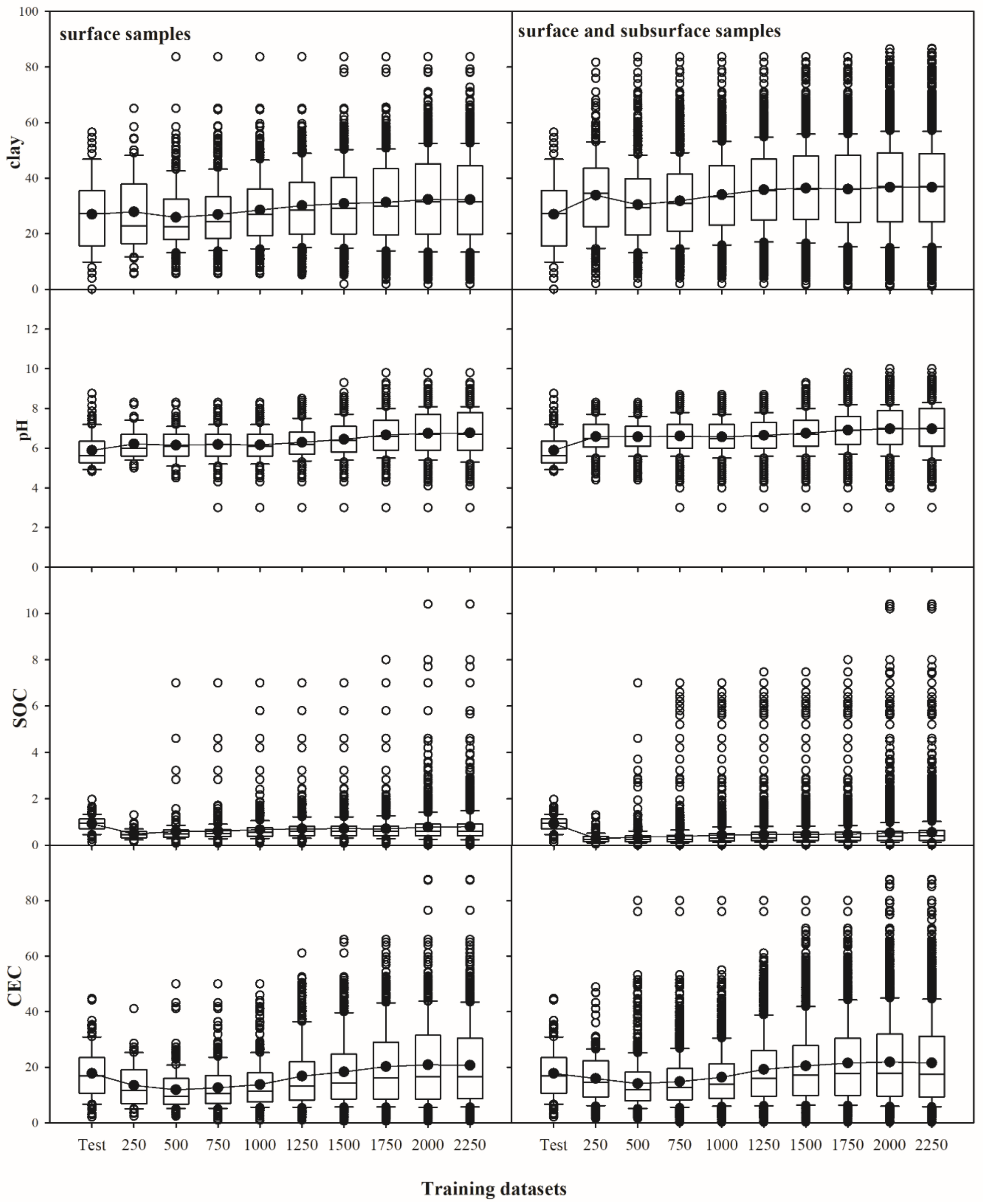

Fig. S2a. Box plots for the clay content, pH, soil organic carbon content (SOC) and cation exchange capacity (CEC) values for the various West Bengal-centric training datasets and the West Bengal test dataset

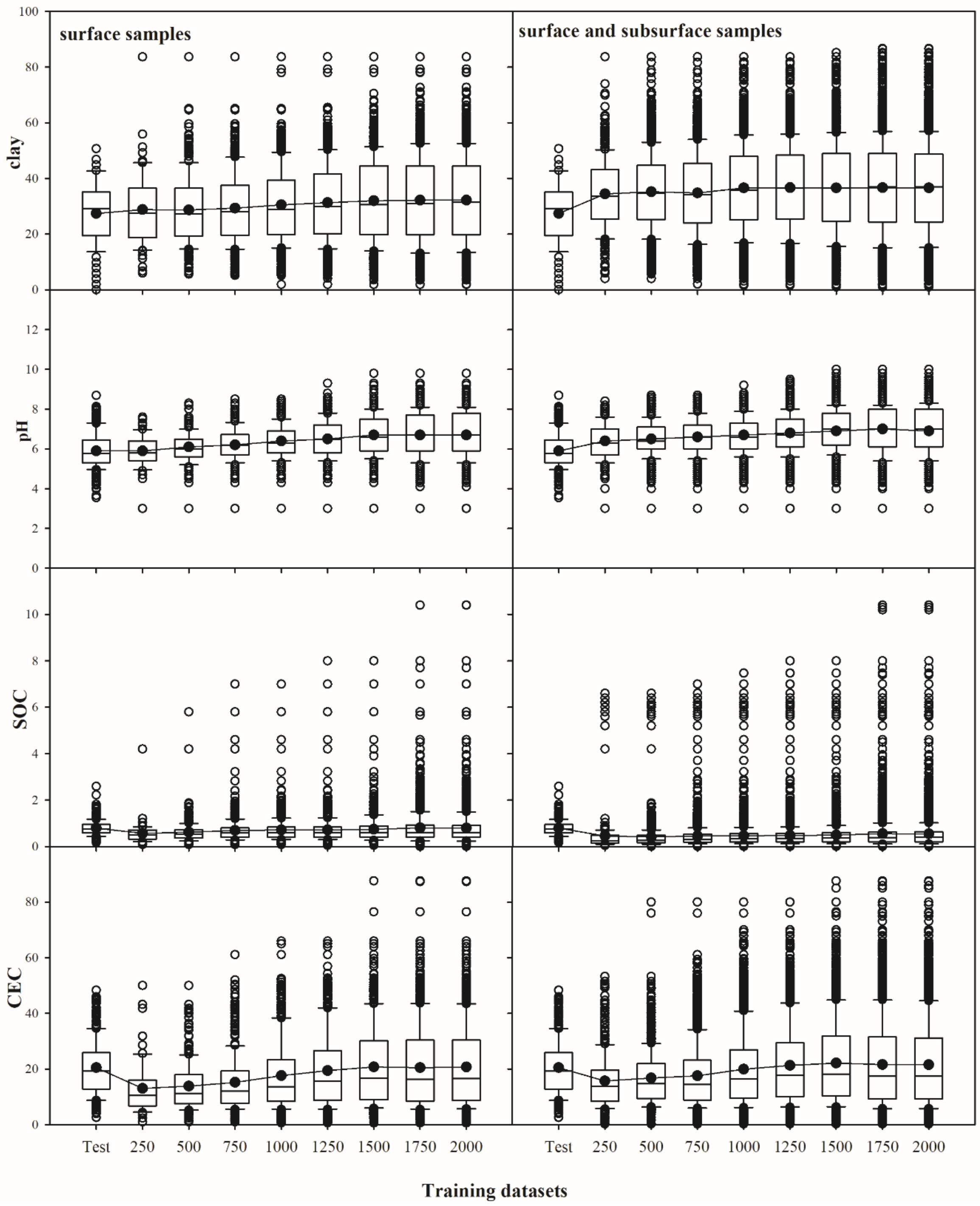

Fig. S2b. Box plots for the clay content, pH, soil organic carbon content (SOC) and cation exchange capacity (CEC) values for the various Odisha-centric training datasets and the Odisha test dataset

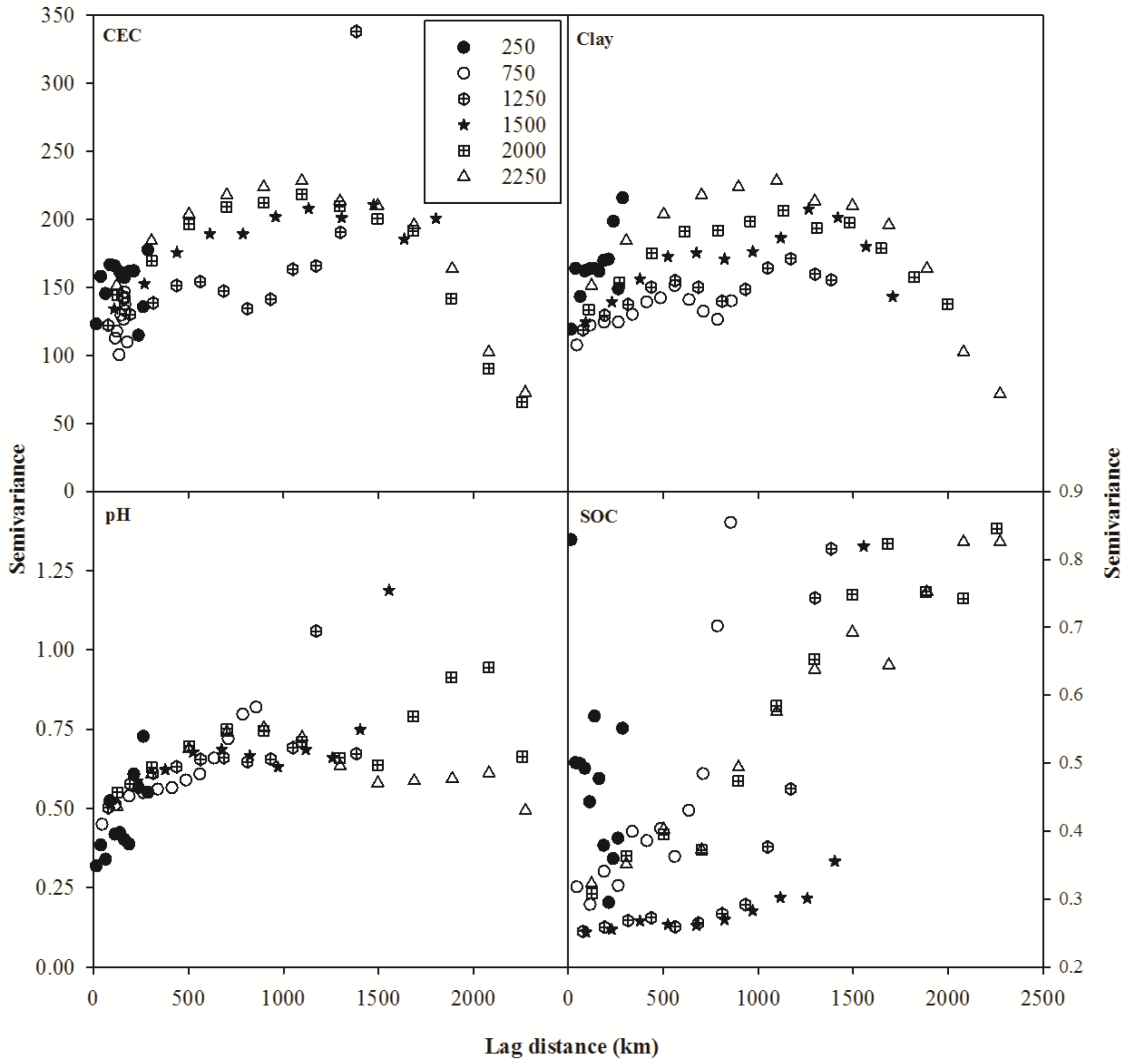

Fig. S3a. Semivariograms for cation exchange capacity (CEC), clay, pH and soil organic carbon (SOC) values for each of the location datasets with radius of 250, 750, 1250, 1500, 2000 and 2250 km from the West Bengal centre of 88.901°E and 23.126°N for surface soil samples.

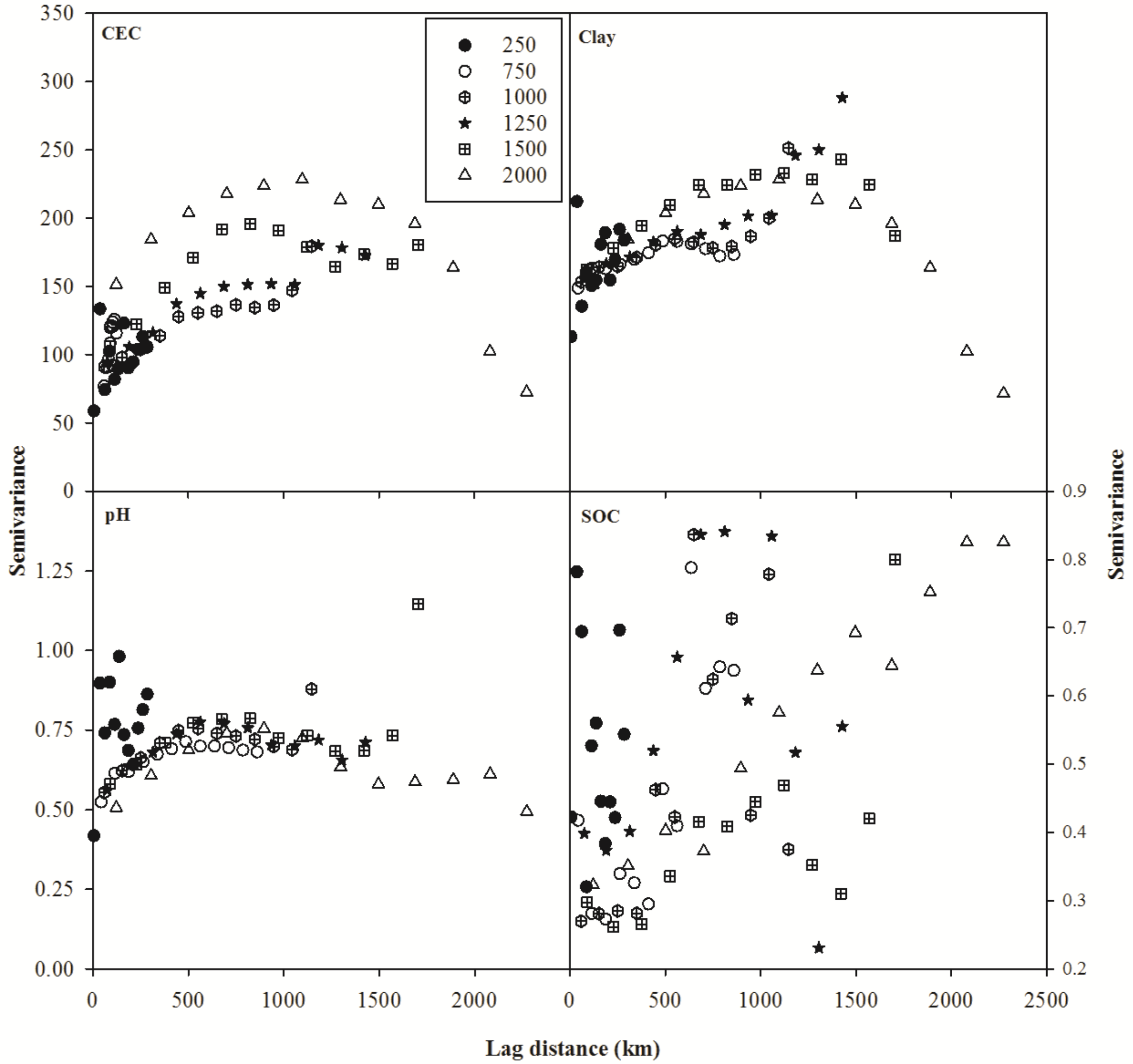

Fig. S3b. Semivariograms for cation exchange capacity (CEC), clay, pH and soil organic carbon (SOC) values for each of the location datasets with radius of 250, 750, 1000, 1250, 1500 and 2000 km from the Odisha centra point of 85.584°E and 21.088°N for whole profile soil samples

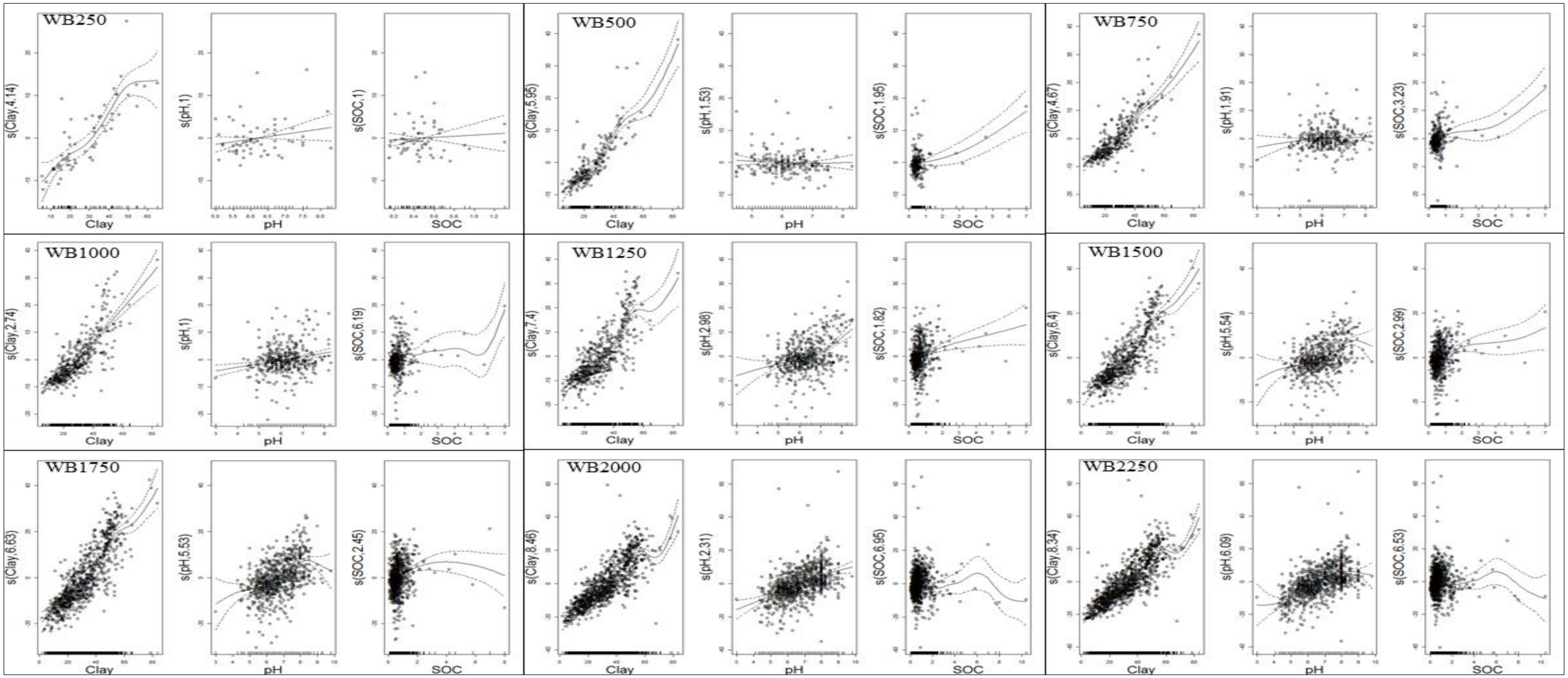

Fig. S4a. Transformations on the predictor variables (clay, clay content; pH; soil organic carbon content, SOC) fitted to a smoothing spline to obtain a generalised additive model for predicting cation exchange capacity (CEC) from clay, pH and SOC for surface soil samples for each location datasets. The effective degrees of spline is given on the y-axis title of each graph. Partial residuals and approximate 95% pointwise confidence bands are shown. WB250 to WB2250 location datasets correspond to soil samples collected from the area enclosed in a circle with radii from 250 km to 2250 km and centre point at 88.901^{o}E and 23.126^{o}N as shown in Fig. 1.

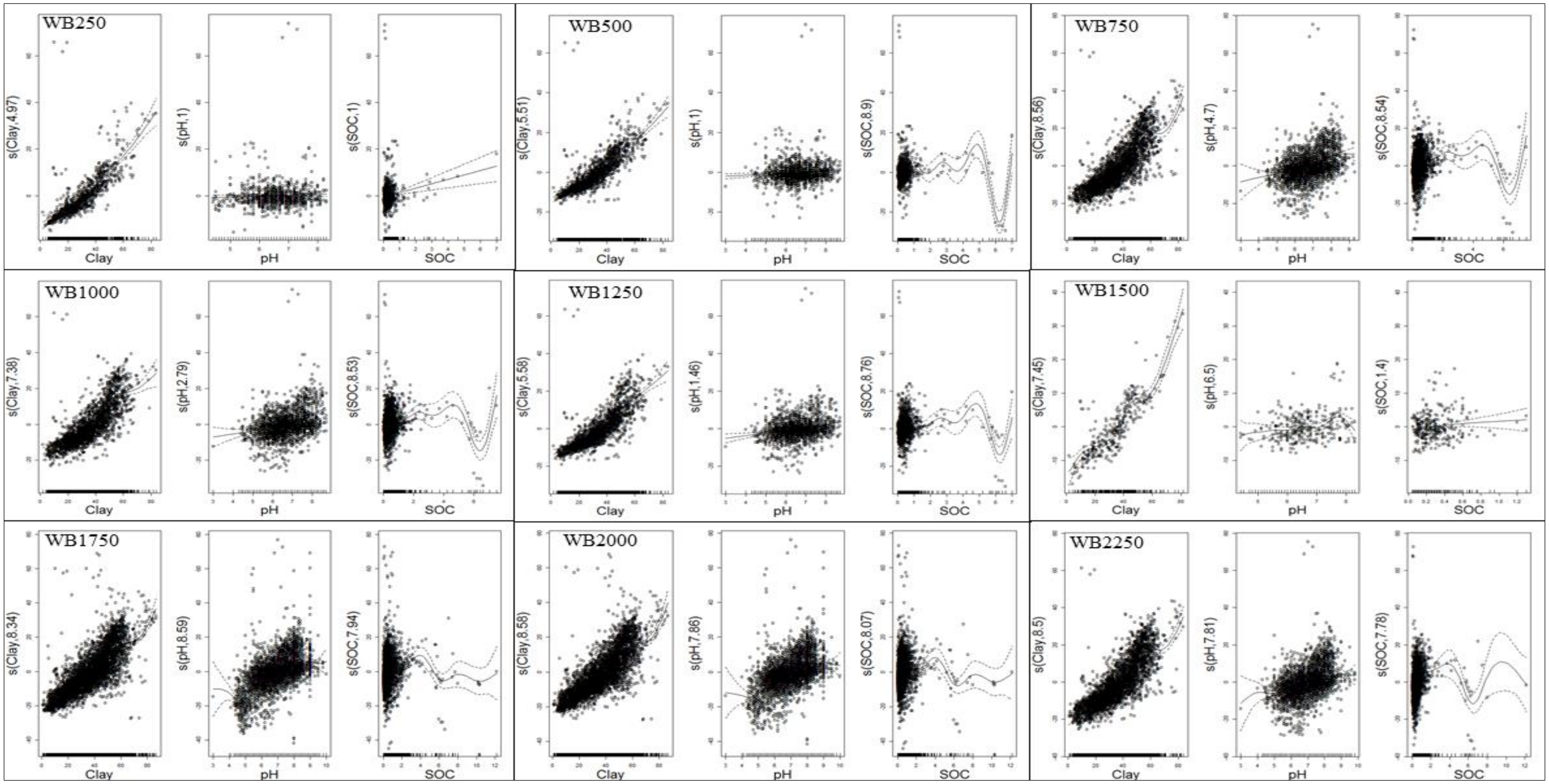

Fig. S4b. Transformations on the predictor variables (clay, clay content; pH; soil organic carbon content, SOC) fitted to a smoothing spline to obtain a generalised additive model for predicting cation exchange capacity (CEC) from clay, pH and SOC for whole profile soil samples for each location datasets. The effective degrees of spline is given on the y-axis title of each graph. Partial residuals and approximate 95% pointwise confidence bands are shown. WB250 to WB2250 location datasets correspond to soil samples collected from the area enclosed in a circle with radii from 250 km to 2250 km and centre point at 88.901^{o}E and 23.126^{o}N as shown in Fig. 1.

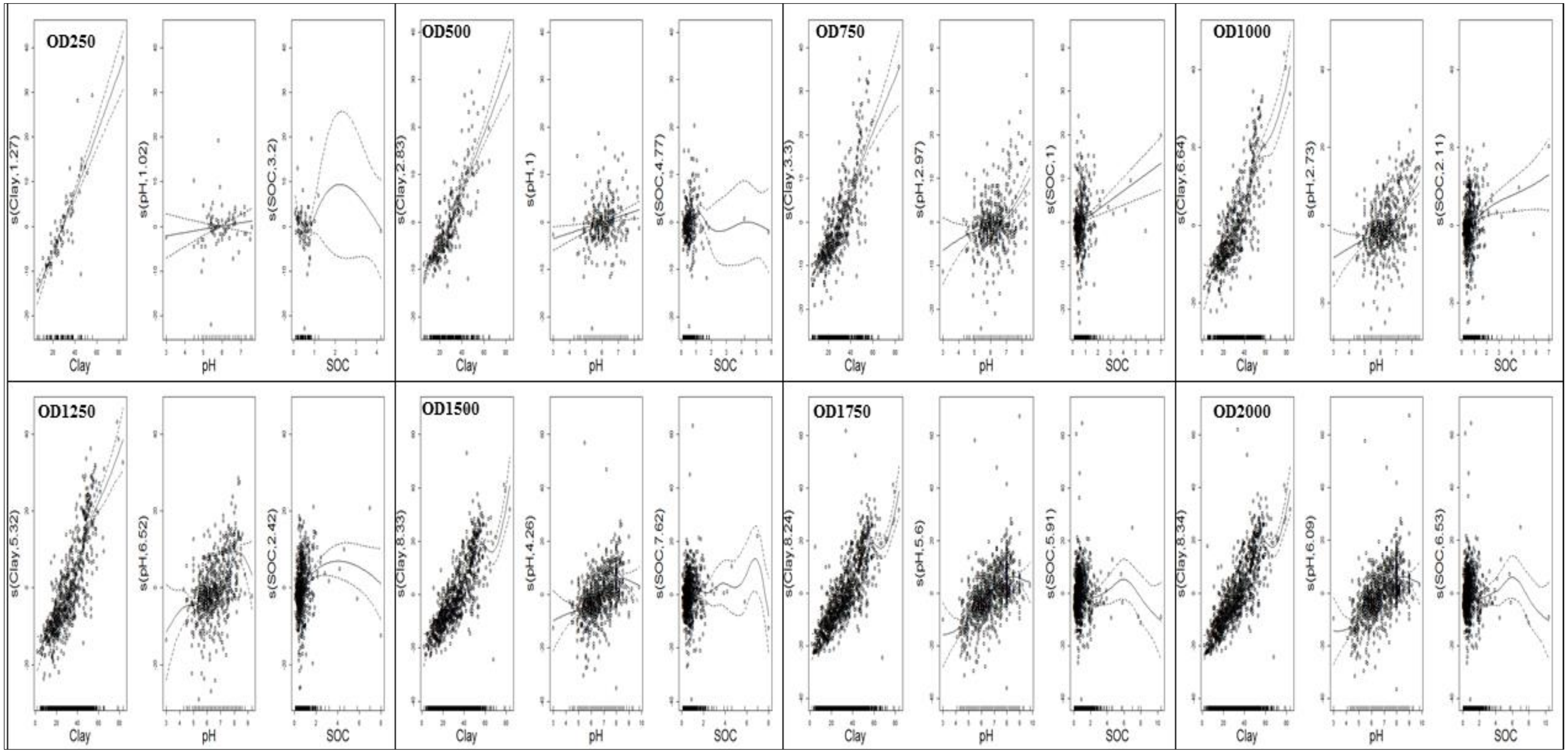

Fig. S4c. Transformations on the predictor variables (clay, clay content; pH; soil organic carbon content, SOC) fitted to a smoothing spline to obtain a generalised additive model for predicting cation exchange capacity (CEC) from clay, pH and SOC for surface soil samples for each location datasets. The effective degrees of spline is given on the y-axis title of each graph. Partial residuals and approximate 95% pointwise confidence bands are shown. OD250 to OD2000 location datasets correspond to soil samples collected from the area enclosed in a circle with radii from 250 km to 2000 km and centre point at 85.584^{o}E and 21.088^{o}N as shown in Fig. 1.

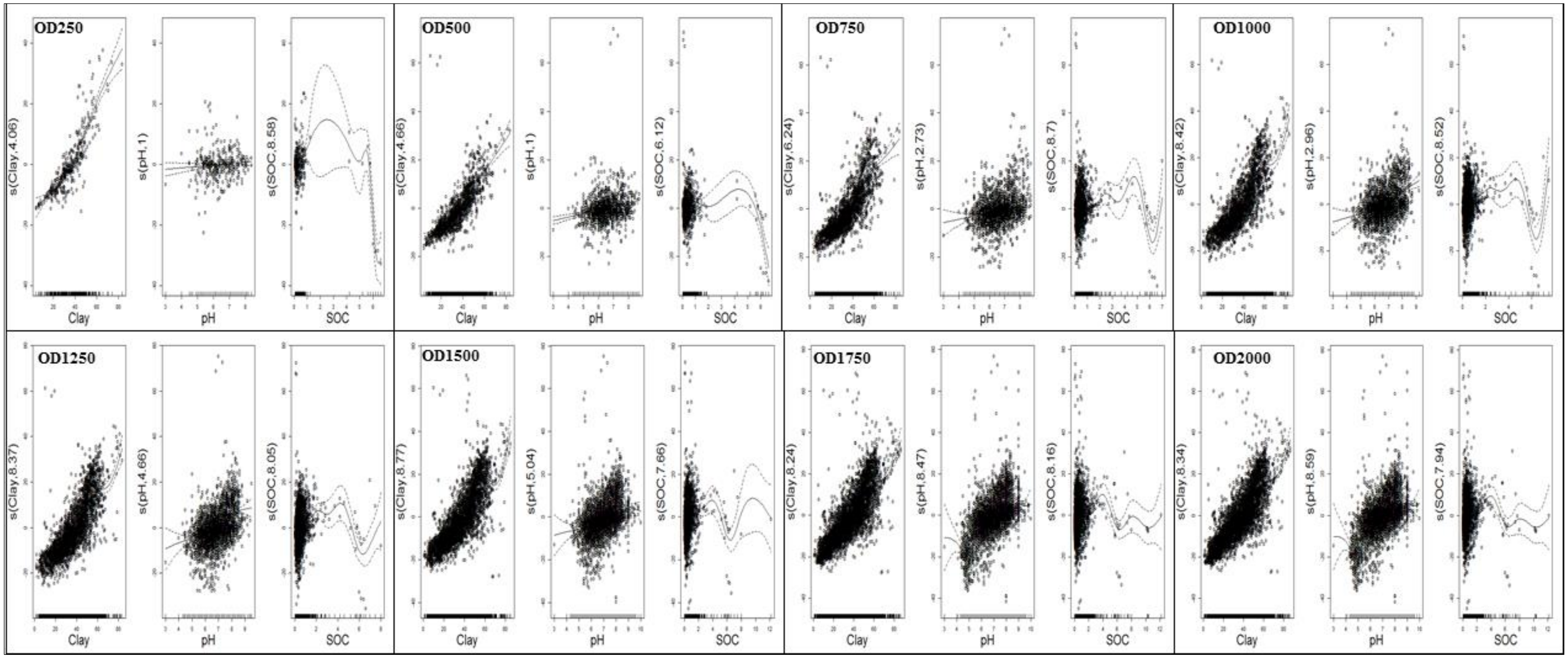

Fig. S4d. Transformations on the predictor variables (clay, clay content; pH; soil organic carbon content, SOC) fitted to a smoothing spline to obtain a generalised additive model for predicting cation exchange capacity (CEC) from clay, pH and SOC for whole profile soil samples for each location datasets. The effective degrees of spline is given on the y-axis title of each graph. Partial residuals and approximate 95% pointwise confidence bands are shown. OD250 to OD2000 location datasets correspond to soil samples collected from the area enclosed in a circle with radii from 250 km to 2000 km and centre point at85.584°E and 21.088°N as shown in Fig. 1.

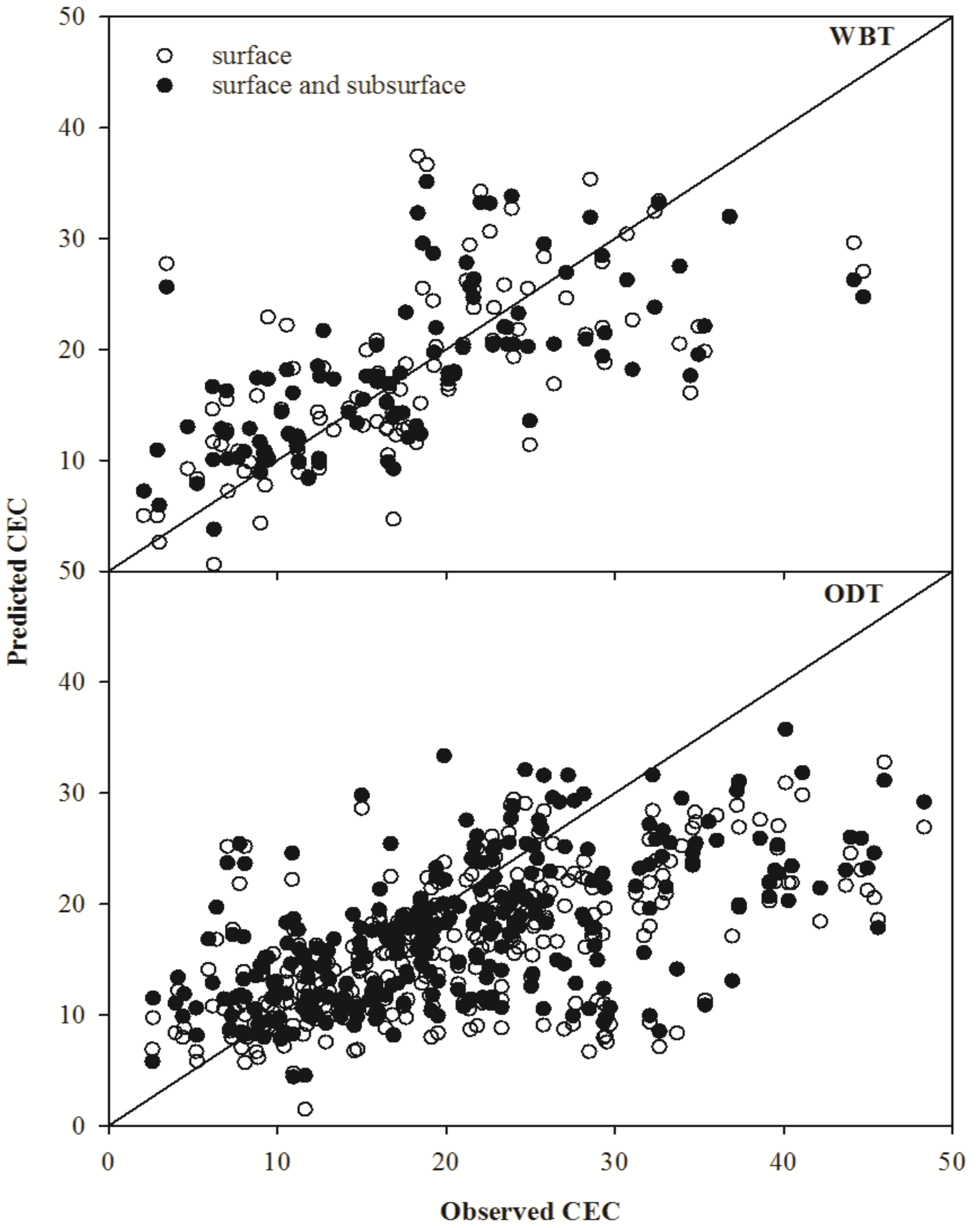

Fig. S5. Observed vs predicted cation exchange capacity (CEC) values by extreme gradient boosting modelling approach trained on the West Bengal-centric Indian soil legacy training datasets WB2000 and WB1750 for West Bengal test (WBT) data and the Odisha-centric soil legacy training dataset OD2000 and OD1500 for Odisha test data (ODT).

Supplementary Tables:

Table S6a. Distance correlation coefficient (dCor) and Pearsons correlation coefficients (ρ) for clay, pH, soil organic carbon (SOC) and clay, pH and SOC combined with CEC for the Indian soil legacy (ISL) location datasets obtained by taking a centre within West Bengal (WB) state

	WB250		WB500		WB750		WB1000		WB1250		WB1500		WB1750		WB2000		WB2250	
**Surface soil samples**																		
	dCor	ρ	dCor	ρ	dCor	ρ	dCor	ρ	dCor	ρ	dCor	ρ	dCor	ρ	dCor	ρ	dCor	ρ
CEC and clay	0.84	**0.83**	0.82	0.83	0.80	0.82	0.79	0.80	0.79	0.80	0.81	0.83	0.81	0.82	0.83	0.80	0.79	0.79
CEC and pH	0.30	0.23	0.12	0.05	0.19	0.15	0.25	0.24	0.43	0.44	0.43	0.45	0.46	0.43	0.47	0.40	0.50	0.40
CEC and SOC	0.40	0.19	0.24	0.15	0.27	0.19	0.27	0.17	0.26	0.14	0.26	0.14	0.22	0.14	0.30	0.05	0.31	0.01
CEC and (clay,pH and SOC)	0.84	NA	0.82	NA	0.80	NA	0.79	NA	0.79	NA	0.81	NA	0.81	NA	**0.84**	NA	0.83	NA
**Whole profile (surface + subsurface) soil samples**																		
	WB250		WB500		WB750		WB1000		WB1250		WB1500		WB1750		WB2000		WB2250	
	dCor	ρ	dCor	ρ	dCor	ρ	dCor	ρ	dCor	ρ	dCor	ρ	dCor	ρ	dCor	ρ	dCor	ρ
CEC and clay	0.81	**0.83**	0.83	0.75	0.83	0.78	0.82	0.78	0.80	0.78	0.80	0.79	0.80	0.79	0.76	0.76	0.73	0.73
CEC and pH	0.26	0.23	0.14	0.09	0.20	0.18	0.26	0.25	0.39	0.39	0.42	0.41	0.42	0.38	0.40	0.37	0.41	0.39
CEC and SOC	0.12	-0.03	0.06	0.00	0.08	-0.02	0.11	0.01	0.15	0.03	0.19	0.05	0.24	0.09	0.20	0.03	0.18	0.01
CEC and (clay,pH and SOC)	0.81	NA	0.83	NA	0.83	NA	0.82	NA	0.80	NA	0.80	NA	**0.84**	NA	0.77	NA	0.73	NA

Table S6b. Distance correlation coefficient (dCor) and Pearsons correlation coefficients (ρ) for clay, pH, soil organic carbon (SOC) and clay, pH and SOC combined with CEC for the Indian soil legacy (ISL) location datasets obtained by taking a centre within Odisha (OD) state

Surface soil samples																
	OD250		OD500		OD750		OD1000		OD1250		OD1500		OD1750		OD2000	
	dCor	ρ	dCor	ρ	dCor	ρ	dCor	ρ	dCor	ρ	dCor	ρ	dCor	ρ	dCor	ρ
CEC and clay	0.83	**0.84**	0.82	0.83	0.79	0.79	0.79	0.81	0.82	0.83	0.81	0.81	0.78	0.81	0.79	0.79
CEC and pH	0.24	0.08	0.36	0.31	0.34	0.35	0.47	0.48	0.50	0.49	0.44	0.40	0.44	0.40	0.50	0.40
CEC and SOC	0.32	0.16	0.30	0.16	0.28	0.15	0.25	0.13	0.24	0.10	0.25	0.11	0.20	0.11	0.31	0.01
CEC and (clay,pH and SOC)	0.83	NA	0.82	NA	0.79	NA	0.79	NA	0.82	NA	0.81	NA	0.79	NA	**0.83**	NA
**Whole profile (surface+ subsurface) soil samples**																
	OD250		OD500		OD750		OD1000		OD1250		OD1500		OD1750		OD2000	
	dCor	ρ	dCor	ρ	dCor	ρ	dCor	ρ	dCor	ρ	dCor	ρ	dCor	ρ	dCor	ρ
CEC and clay	0.81	**0.80**	0.82	0.76	0.80	0.77	0.80	0.78	0.80	0.78	0.78	0.78	0.74	0.73	0.73	0.73
CEC and pH	0.20	0.09	0.37	0.34	0.32	0.32	0.42	0.18	0.45	0.39	0.39	0.36	0.41	0.39	0.41	0.39
CEC and SOC	0.15	-0.09	0.09	-0.02	0.15	0.02	0.18	-0.02	0.21	0.03	0.22	0.08	0.19	0.02	0.18	0.01
CEC and (clay,pH and SOC)	0.81	NA	0.82	NA	0.80	NA	0.80	NA	0.80	NA	**0.82**	NA	0.74	NA	0.73	NA

Table S7a. Number of samples, soil orders, and the agro-ecological zone characteristics of Indian soil legacy (ISL) location datasets. WB250 to WB2250 location datasets correspond to soil samples collected from the area enclosed in a circle with radii from 250 km to 2250 km and centre point at 88.901^{o}E and 23.126^{o}N as shown in Fig. 1.

Location datasets	Number of samples		Soil Orders	Agro-ecological Regions
	Whole Profile	Surface		
WB250	301	62	Alfisols, Inceptisols, Entisols	Warm perhumid eco-region with red hill soils and hot, subhumid to semiarid with coastal alluvium-derived soil
WB500	909	199	Alfisols, Inceptisols, Entisols	Same as WB500 plus hot subhumid ecoregion with alluvium derived soil and red and laterite soils
WB750	1470	328	Alfisols, Inceptisols, Entisols, Mollisols, Vertisols	Same as WB500
WB1000	1820	418	Alfisols, Inceptisols, Entisols, Mollisols, Vertisols	Same as WB500 plus hot semiarid region with shallow and medium black soil
WB1250	2271	544	Alfisols, Inceptisols, Entisols, Mollisols, Aridisols, Vertisols	Same as WB1000 plus hot semiarid region deep black soil and loamy soil
WB1500	2617	643	Alfisols, Inceptisols, Entisols, Mollisols, Aridisols, Vertisols	Same as WB1250
WB1750	3106	812	Alfisols, Inceptisols, Entisols, Mollisols, Aridisols, Vertisols	Same as WB1250 plus hot arid ecoregion with desert and saline soils
WB2000	3810	1000	Alfisols, Inceptisols, Entisols, Mollisols, Aridisols, Vertisols, Ultisols	Same as WB1750
WB2250	4190	1092	Alfisols, Inceptisols, Entisols, Mollisols, Aridisols, Vertisols, Ultisols	Same as WB1750 plus hot, humid to perhumid regions with red, lateritic and alluvium derived soils

Table S7b. Number of samples, soil orders, and the agro-ecological zone characteristics of Indian soil legacy (ISL) location datasets. OD250 to OD2000 location datasets correspond to soil samples collected from the area enclosed in a circle with radii from 250 km to 2000 km and centre point at 85.584^{o}E and 21.088^{o}N located in Odisha as shown in Fig. 1.

Location datasets	Number of samples		Soil Orders	Agro-ecological Regions
	Whole Profile	Surface		
OD250	339	73	Alfisols, Inceptisols, Entisols, Aridisols	Hot subhumid red and laterite soils
OD500	1280	287	Alfisols, Inceptisols, Entisols, Aridisols	Same as OD500 plus hot subhumid ecoregion with alluvium derived soil and red and laterite soils
OD750	2014	466	Alfisols, Inceptisols, Entisols, Mollisols, Vertisols, Aridisols	Same as OD500
OD1000	2395	571	Alfisols, Inceptisols, Entisols, Mollisols, Vertisols, Aridisols	Same as OD500 plus hot semiarid region with shallow and medium black soil
OD1250	2797	696	Alfisols, Inceptisols, Entisols, Mollisols, Aridisols, Vertisols	Same as OD1000 plus hot semiarid region deep black soil and loamy soil
OD1500	3438	884	Alfisols, Inceptisols, Entisols, Mollisols, Aridisols, Vertisols	Same as OD1250
OD1750	4028	1030	Alfisols, Inceptisols, Entisols, Mollisols, Aridisols, Vertisols	Same as OD1250 plus hot arid ecoregion with desert and saline soils
OD2000	4190	1092	Alfisols, Inceptisols, Entisols, Mollisols, Aridisols, Vertisols, Ultisols	Same as OD1750 plus hot, humid to perhumid regions with red, lateritic and alluvium derived soils

Supplementary methods

Modelling algorithms used

Five different supervised regression algorithms were used to develop PTFs for CEC. Briefly, the MLR is a statistical technique that uses several explanatory variables to predict the outcome of a response variable (Moore et al., 1993). It fits an observed response data set (e.g., CEC) using a linear combination of independent predictors (e.g., pH, clay and SOC contents). Ridge regression is a shrinkage method for MLR. It is a technique for analysing multicollinearity in regression data. When multicollinearity occurs, least squares estimates are unbiased, but their variances are large so they may be far from the true value. By adding a degree of bias to the regression estimates, ridge regression reduces the standard errors. Support Vector Machine (SVM) is a novel machine-learning tool that has been originated from Statistical Learning Theory developed by Vapnik in 1995[1].Selection of optimal SVM parameters is the important step in SVM design. For the PTF developed in this study, we have considered a RBF kernel function and used genetic algorithm (GA) to optimize regularization parameter C, bandwith of RBF kernel σ^2 and radius of a tube loss function ε. Details of the SVM and how GA is used to optimize the parameters may be found in Sajan et al. (2015). Random forests (RF) follows a bagging algorithm. In Bagging, successive models do not depend on earlier models and each model is independently constructed using a bootstrap sample of the observation dataset[3]. Breiman (2001) proposed random forests, which add an additional layer of randomness to bagging. In a random forest, each node is split using the best among a subset of predictors randomly chosen at that node. It works by variance reduction through averaging; however, the bias remains unaltered.N_estimators, which represents the number of trees in the forest and max_depth, which represents the depth of each tree in the forest was tuned for the PTFs

developed based on leave-one-out cross validation of the training dataset. The XGB algorithm is a boosting algorithm. Boosting is based on the theorem “The Strength of Weak Learnability”[5]. It is an ensembling technique where we build many independent models and combine them using model averaging technique. The observations in boosting algorithm are chosen based on the error in a sequential manner. Each new model is a fit on a modified version of the original data set and each subsequent model aims to reduce the errors of the previous model. Therefore, the observations have an unequal probability of appearing in subsequent models and ones with the highest error appear most. Gradient boosting involves three elements: a loss function to be optimized, a weak learner to make predictions, and an additive model to add weak learners to minimize the loss function.If we consider a response variable Y, predictor variables x_1, x_2, …, x_p, and a loss function L (Y, *f*), the gradient boosting algorithm (Friedman, 2001) is given as:

1. $F_o(x) = \arg\min_\rho \sum_{i=1}^{N} L(Y_i, \rho)$
2. For m =1 to M, do
   a. $\tilde{Y}_i = -\left[\frac{\partial L(Y_i, F(x_i))}{\partial F(x_i)}\right]_{F(x)=F_{m-1}(x)}, i = 1, ..., N$

   b. $a_m = \arg\min_{a,\beta} \sum_{i=1}^{N} \left[\tilde{Y}_i - \beta h(x_i; a)\right]^2$

   c. $\rho_m = \arg\min_\rho \sum_{i=1}^{N} L(Y_i, F_{m-1}(x_i) + \rho_m h(x_i; a_m))$

   d. $F_m(x) = F_{m-1}(x) + \nu.\rho_m h(x; a_m)$
3. End For Loop

   End Algorithm

where ν is a shrinkage factor. If we consider the *h(x,a)* to be a tree, *a* would parameterize the split, ρ is the learning rate. The regression tree *h(x,a)* is the weak learner in the algorithm and fits to the residuals of step 1. A negative gradient shows the downhill direction of the loss function. The Loss function must be differentiable (for example, squared error or absolute error). A fixed number of trees are added or training stops once loss reaches an acceptable level or no longer improves on an external test dataset. Gradient boosting is a greedy algorithm and can over fit a training dataset quickly. It can benefit from regularization methods that penalizes various parts of the algorithm and generally improve the performance of the algorithm by reducing overfitting. The XGB is an efficient and scalable tree based implementation of gradient boosting framework[6, 7, 8]. It drives fast learning through parallel and distributed computing and offers efficient memory usage. For our dataset, we tuned the learning rate eta, the maximum tree depth, fraction of observation to be randomly samples, and fraction of column to be randomly sampled for each tree using a leave one out cross validation of the training datasets. We used the R packages *xgboost, e1071*, *randomForest*, *glmnet* to implement the above modeling approaches with our datasets.

References

1. Corrina C. & V. Vladimir. Support vector networks. *Machine Learning*, 20, 273-297 (1995).
2. Sajan, K. S., Kumar, V., & Tyagi, B. Genetic algorithm based support vector machine for on-line voltage stability monitoring. Int. J. Elec. Power, 73, 200-208 (2015).
3. Breiman, L. Bagging predictors. *Machine learning*, 24(2), 123-140 (1996).
4. Breiman, L. Random forests. *Machine learning*, 45(1), 5-32 (2001).
5. Schapire, R. E. The strength of weak learnability. *Machine learning*, 5(2), 197-227 (1990).
6. Friedman, J. H. Greedy function approximation: a gradient boosting machine. *Annals of statistics*, 1189-1232 (2001).
7. Friedman, J., Hastie, T., &Tibshirani, R. Additive logistic regression: a statistical view of boosting (with discussion and a rejoinder by the authors). *The annals of statistics*, 28(2), 337-407 (2000).

8. Chen, T., He, T., Benesty, M., Khotilovich, V., & Tang, Y. Xgboost: extreme gradient boosting. R package version 0.4-2, 1-4 (2019).
